# Basic swimming or water safety skills training for drowning prevention in children: an updated systematic review

**DOI:** 10.3389/fpubh.2025.1698353

**Published:** 2025-12-12

**Authors:** Yana Criel, Pieter Severijns, Hans Scheers, Emmy De Buck

**Affiliations:** 1 Centre for Evidence-Based Practice, Belgian Red Cross-Flanders, Mechelen, Belgium; 2 Department of Public Health and Primary Care, Leuven Institute for Healthcare Policy, KU Leuven, Leuven, Belgium; 3 Cochrane First Aid, Mechelen, Belgium

**Keywords:** drowning, swimming skills, water safety, systematic review, children

## Abstract

**Background:**

Child drownings constitute a major cause of mortality from unintentional injury worldwide. In 2021, the WHO issued a strong recommendation in favour of basic swimming skills and water safety training in children to prevent drowning. The systematic review conducted in support of this guideline, however, revealed evidence of overall very low certainty and illustrated a number of research gaps. Here, the evidence from this systematic review was updated.

**Methods:**

Five scientific databases (PubMed, Embase, The Cochrane Library, Web of Science, ERIC) and two clinical trial registers (clinicaltrails.gov, WHO ICTRP) were searched for controlled experimental and observational studies. Interventions delivering basic swimming skills or water safety in children under the age of 18 years were included. Outcomes of interest included drowning-related mortality and morbidity, and water safety skills, knowledge and behaviour. Certainty of the evidence was appraised using the GRADE methodology.

**Results:**

A total of 33 studies were included, of which 21 were previously included in the original review and 12 were newly identified in the current update. Studies delivered either basic swimming skills (18 studies), isolated water safety training (6 studies), water safety training as part of an injury prevention programme (5 studies) or an intervention combining swimming and water safety (4 studies). Certainty of the evidence ranged from high to very low.

**Conclusion:**

Swim training may reduce drowning-related mortality and increase water safety skills in children. The used pedagogical approach (focus on familiarization and motor awareness), mode of delivery (using video-taped feedback) and setting (shallow water) may be determining factors for swimming skills acquisition. Water safety training is effective to enhance knowledge of and safe behaviour in/around water, and may reduce drowning mortality. These results, highlighting the importance of promoting swimming and water safety skills from an early age onward, could inform public health strategies and guide the development of sustainable interventions in communities at high risk of drowning.

**Systematic review registration:**

PROSPERO registration CRD42020167437; https://www.crd.york.ac.uk/PROSPERO/view/CRD42020167437.

## 1 Introduction

Drowning is one of the leading causes of mortality from an unintentional injury worldwide. According to a recent estimation of the World Health Organization (WHO), 300,000 fatal drownings occurred in 2021 (1). In particular children growing up in low-and middle-income countries (LMIC) are heavily impacted (2, 3). Children between the ages of 1–4 years and 5–14 years account for approximately 24% and 19% of the reported drowning deaths, respectively. Over 90% of fatal submersion injuries take place in LMICs (1, 67).

Over the last 20 years, there has been a growing commitment to increasing global awareness on the burden of drowning and pushing for governmental action to reduce drowning mortality. In 2008, the WHO and UNICEF World Report on Child Injury Prevention (4) was published, summarizing evidence-based drowning prevention strategies. In 2014, the WHO further advocated for research, community-based actions, and policy and legislation changes in the Global Report on Drowning (67). These continued efforts have likely contributed to the gradual decline in global drowning mortality and drowning death rate from 2000 to 2019 (1). Nevertheless, unintentional drowning numbers remain alarmingly high, especially in low-resource settings. Moreover, an increase in the magnitude and frequency of flooding events is anticipated as a consequence of the active climate crisis (5). These observations urge for continued multi-domain, evidence-based actions to ensure the continuation of the positive tendency.

Several scoping and systematic reviews have synthesized evidence on the effectiveness of public health interventions aimed at reducing drowning incidents (6–11). Commonly investigated prevention strategies, though with varying levels of effectiveness, include: setting up barriers or fences around bodies of water, establishing safe areas for children to play away from a body of water, ensuring children are supervised by an adult, providing rescue and resuscitation training to laypeople, and teaching children basic swimming skills and water safety. Especially the latter stands out as an important avenue to explore. That is, most drowning prevention interventions depend on external factors which may not be reliably available (e.g., adult presence, safe infrastructure), in particular in low-resource settings. Equipping children themselves with the skills to recognize and respond to water-related risks may offer a more direct and potentially more sustainable prevention strategy. Nevertheless, the effectiveness of these interventions to prevent drowning has not been conclusively established, partly due to the large heterogeneity in the populations and interventions studied.

Based on a systematic review on drowning prevention interventions, Ashraf et al. (6) concluded that teaching swimming, water safety and safe rescue skills in children over the age of 6 years is a promising protective strategy for drowning. However, conclusive proof for the effectiveness of these interventions was lacking. Synthesizing the evidence in adolescents between the ages of 10 and 14 years, Olivar et al. (12) could not demonstrate a clear association between children’s swimming skills and the risk of fatal drowning. In addition, there are still open questions related to the impact of how swimming skills interventions are delivered (e.g., pedagogical approach, didactic materials, setting, training frequency) on the effectiveness of these training programmes. Mekkaoui et al. (13) summarized the available evidence on adequate pedagogical strategies for enhancing aquatic literacy in children aged 4 to 6 years. While there was some evidence suggesting that incorporating environmental constraints (i.e., teaching in shallow water, using video feedback) may be beneficial in swim training, the key takeaway was a lack of agreement on which approach is more favourable due to the limited amount of high-quality evidence and the large heterogeneity of pedagogical characteristics studied.

In 2019, we conducted a comprehensive systematic review to inform the development of the WHO Guideline on drowning prevention (14). Although the results of this review were included in the published WHO guideline document, the systematic review itself was not released as a stand-alone peer-reviewed publication. This review targeted the effect of basic swim and/or water safety training in children of any age on drowning-related mortality and morbidity, and on water safety skills, knowledge and behaviour. Drawing on the available evidence, ranging from moderate to very low certainty, the guideline development group issued a strong recommendation in favour of basic swimming skills and water safety programmes for the prevention of submersion injuries in low-, middle- and high-income countries. Nevertheless, the review revealed a number of gaps in the available research, including (1) the underrepresentation of LMIC in the current body of research, (2) the limited reporting on direct outcomes on drowning-related mortality and morbidity, (3) the need for evidence on best implementation practices for swimming skills and water safety programmes (e.g., pedagogical approach, programme duration), and (4) the failure to consider adverse events. To further address these gaps and considering that systematic reviews are typically deemed outdated 5 years after their search date (15), this study sought to update the 2019 systematic review. The strength of this review lies in its broad focus, both in terms of the interventions studied (i.e., swim training and water safety education), as well as the study population (i.e., all children under the age of 18 years). In contrast, previously discussed reviews often provide a more fragmented view by limiting their scope to a specific age group or intervention.

Specifically, this investigation aimed to update the evidence base on the effectiveness of educational programmes that provide basic swimming skills and/or water safety training *(Intervention)* to children of any age *(Population)* compared to no such programmes or other drowning prevention approaches *(Comparator)* in reducing drowning-related mortality or morbidity and/or increasing water safety knowledge, skills or behaviour *(Outcome)*. The primary objective of this review was to assess whether swimming and water safety interventions are effective in reducing drowning mortality and morbidity in children. The secondary aim was to identifying key characteristics of such programmes that determine its effectiveness in improving children’s water safety knowledge, skills and behaviours.

## 2 Methods

This review was registered on PROSPERO (CRD42020167437). The methodology follows the Centre for Evidence-Based Practice methodological charter (66) and reporting adheres to the Preferred Reporting Items for Systematic Reviews and Meta-analysis (PRISMA) 2020 checklist (Appendix 1) (68).

### 2.1 Selection criteria

#### 2.1.1 Publication type and study design

Interventional and observational studies with a controlled design, specifically randomized controlled trials (RCT), quasi- or non-RCTs, controlled before-after studies, controlled interrupted time series, cohort studies and case–control studies, were included. Systematic reviews and scoping reviews were screened for potentially relevant studies.

Eligible publication types were peer-reviewed articles, scientific letters, letters to the editor, governmental reports, policy documents and dissertations. Peer-reviewed protocols and clinical registrations were included if the data were available. Conference abstracts or conference proceedings were included if data were available and not covered by a peer-reviewed article. In the absence of data or a peer-reviewed report, these publications were labelled *awaiting classification*.

#### 2.1.2 Population

Intervention studies in children and adolescents, aged 0–17 years, were included. If both participants under and over the age of 18 years were included in the study sample, the study was only considered in case a separate analysis was available for under 18 year-olds. Interventional studies targeted at children or adolescents with a chronic illness, a developmental disorder, or a physical/intellectual disability were not included.

#### 2.1.3 Intervention and comparator

Interventions provided to children comprising an educational program, delivering either basic swimming skills, water safety training or a combination, were eligible for inclusion. Basic swimming skills training was defined as any intervention delivering either basic aquatic competences (i.e., breath control, floating, kicking, diving, coordination, self-rescue) or basic swimming strokes (i.e., breaststroke, backstroke, front crawl) to children with no or limited previous formal swimming education through in-water practice. Water safety training included any out-of-water intervention aimed at delivering knowledge or skills related to safe behaviour in and around water (e.g., running in the vicinity of a swimming pool, entering a body of water without adult supervision). Multi-component injury prevention or home safety programs were included if water safety education was part of the program. Programs exclusively directed at parents or caregivers were excluded. The educational programs could be delivered via in-class training, out-of-class training near a body of water, in-water training in a swimming pool or body of water, or a combination of these. Interventions including only an informal component without in-person teaching (e.g., posters, flyers) were excluded.

Comparisons of interest for basic swimming skills programmes (in-water) included (1) no intervention, (2) an at least partially in-water variant of the basic swimming skills intervention, (3) an educational programme for water safety (out-of-water), and (4) any other drowning prevention intervention. Comparisons of interest for water safety training programmes (out-of-water) included (1) no intervention, (2) a variant of the water safety intervention (out-of-water), and (3) any alternative drowning prevention intervention.

#### 2.1.4 Outcomes

Drowning-related mortality (i.e., fatal drownings), drowning-related morbidity (i.e., non-fatal drownings followed by aspiration pneumonia, brain damage or organ damage, and potential death within 1 month of drowning), and total number of drowning accidents (i.e., fatal or non-fatal submersion in water for which rescue is required, with or without subsequent morbidity) were the primary outcomes. Secondary outcomes included measures of water safety knowledge, water safety skills, water safety behaviour, programme safety and cost-effectiveness.

### 2.2 Data sources and study selection

MEDLINE, PMC and NCBI bookshelf (via the PubMed interface), Embase (via the Embase.com interface), the Cochrane Library (CDSR and CENTRAL), Web of Science, and ERIC (via the OvidSP interface) were searched. To identify unpublished and ongoing studies, Clinicaltrials.gov and the WHO International Clinical Trials Registry Platform (ICTRP) were additionally consulted. Database searches, provided in Appendix 2, were run on 13 February 2025. The searches were adopted from the original systematic review (14). A date filter, starting from 1 September 2019, was applied to obtain a two-month overlap with the previous search, run on 17 November 2019.

Records retrieved based on the searches were imported in Endnote. Duplicates were removed using the automatic duplicate detection functionality, followed by a manual verification by one of the reviewers (PS). Records were screened by two independent reviewers (PS and YC), first based on title and abstract, followed by a full text screening in a second stage. After independent screening, a consensus was reached between the reviewers, either through discussion or through consultation of a third reviewer (EDB).

For all included studies (both studies included based on the 2019 search and studies included in the current update), reference lists and the 20 first similar articles in PubMed were screened to identify potentially relevant records. In addition, several sources of grey literature were searched for terms relating to drowning, swimming, water safety, water skills and water training. An overview of these sources, screened on 17 March 2025, is available in Appendix 2.

### 2.3 Data extraction

Two reviewers (PS and YC) independently extracted study characteristics and data from the included studies in *a priori* developed extraction forms. In case of insufficient information, study authors were contacted for additional details. If multiple reports on a single study were available, data were merged under one study ID.

Continuous data were presented as mean differences with 95% confidence interval (CI). Dichotomous data were presented as (adjusted) risk ratios or (adjusted) odds ratios with 95% CI. For non-randomized studies reporting both unadjusted and adjusted effects, only the adjusted data were extracted (16). If effect estimates were not reported in the study, whenever possible, these were calculated in Review Manager 5.4. Drowning mortality rates (i.e., the number of drowning deaths per 100,000 individuals per year) were expressed as rate ratios with 95% CI.

### 2.4 Risk of bias and grading of the certainty of the evidence

Two reviewers (PS and YC) independently determined the risk of bias of the included studies at the outcome level. Randomized controlled trials were appraised using the Cochrane RoB-2 tool for either parallel or cluster-randomized trials (17). For non-randomized experimental or observational studies the ROBINS-I tool (18) was used. Risk of bias was visualized using the robvis tool (19). Certainty of evidence was assessed for each outcome using the Grading of Recommendations, Assessment, Development and Evaluation (GRADE) methodology (20).

### 2.5 Data synthesis

As a result of the large heterogeneity in populations, interventions and outcomes across studies, meta-analyses, sensitivity analyses or subgroup analyses could not be performed. Data were synthesized narratively and the magnitude of the effect and the certainty of the evidence was considered in formulating the evidence conclusions.

### 2.6 Deviations from the intended protocol

In view of the large amount of and considerable heterogeneity in the outcomes measured, this review focussed on outcomes measured at the end of an intervention programme and at follow-up measurements. Data from mid-intervention measurements were omitted.

## 3 Results

### 3.1 Search results

**Figure 1:**
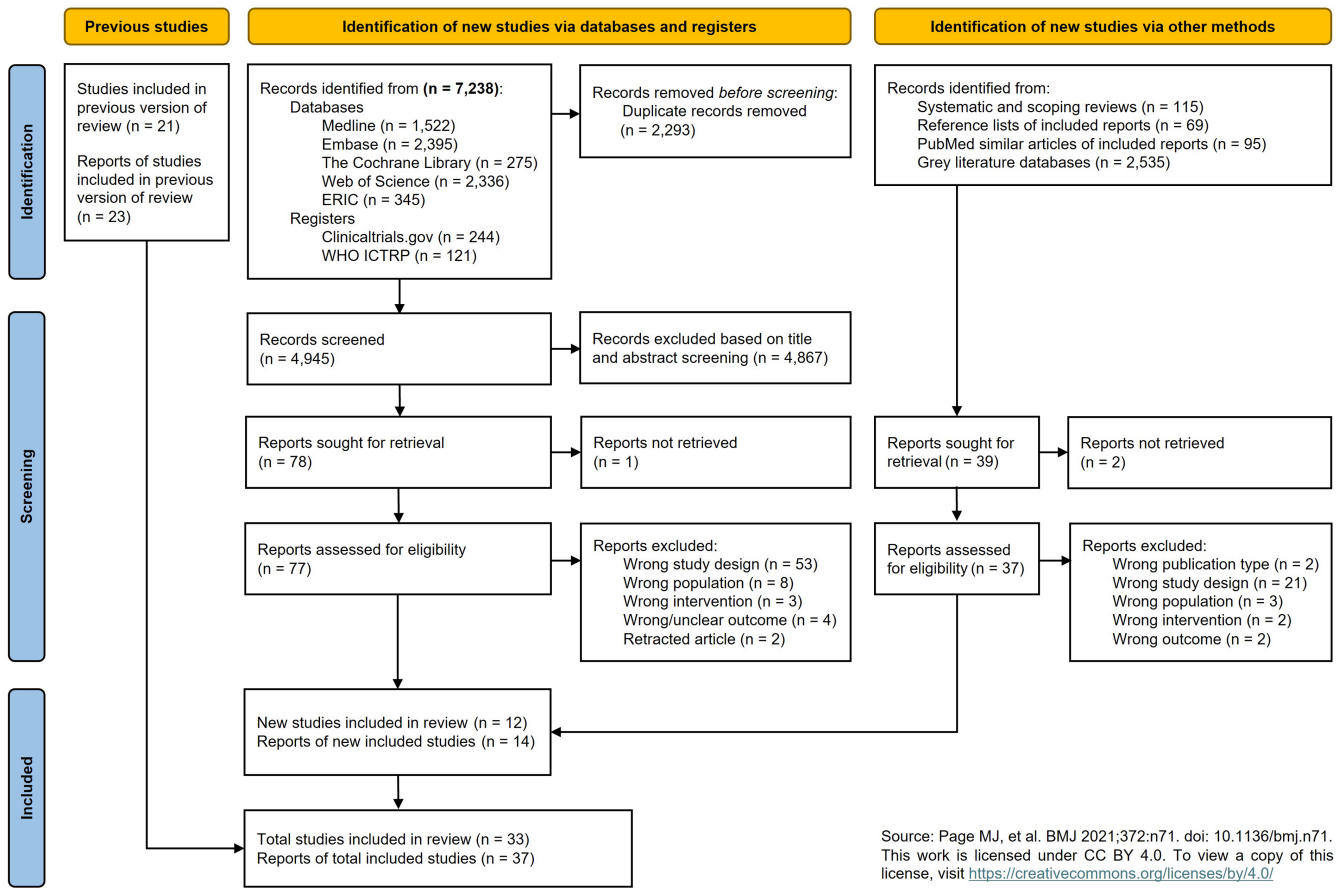
Prisma flowchart of the study selection process.

A detailed overview of the study selection process is provided in Figure 1. In 2019, the search yielded 22 reports from 21 studies. Upon rerunning the updated search in the databases and registers, a total of 7,238 records were identified. After duplicate removal, this number was reduced to 4,945 records for screening. Based on the screening process, 7 reports were found eligible for inclusion. An additional 7 reports were identified via alternative search methods (i.e., 5 from the reference list of systematic and scoping reviews, 2 via the similar articles in PubMed). These 14 records, identified in the current review update, reported on 12 new studies. Combined with the 21 studies from the original review, this resulted in a total of 33 included studies. Appendix 3 provides details of the studies awaiting classification. Below, we report the results of the complete body of evidence (33 studies).

### 3.2 Study characteristics

An overview of the characteristics of the 33 studies included in this review is provided in Table 1. We included thirteen (cluster- or parallel) randomized controlled trials, twelve non-randomized controlled trials, five retrospective cohort studies and three case–control studies. The majority of studies (*n* = 23) were conducted in high income countries (HIC). Ten studies were set up in low- and middle-income countries (LMIC). Interventions were mainly targeted at children under the age of 6 years (*n* = 11) or elementary school children (between the age of 6 and 12 years) (*n* = 16). One study specifically targeted a group of adolescents (>12 years). Four studies included both elementary school children and adolescents, while two observational studies included both children under the age of 6 years and elementary school children.

**Table 1:** Characteristics of included studies.

Study ID, country	Design	Participants^a^	Relevant comparison		Intervention duration	Relevant outcomes
			Intervention/risk factor	Control		
Araiza-Alba et al., 2021 (40), Australia	Experimental: RCT	*N* = 115 Intervention 1: 46, intervention 2: 28, control: 41 Sex: 48 male, 67 female Age (range): 10–12 years	1. Water safety training (out-of-water) using 360° virtual reality 2. Water safety training (out-of-water) using traditional videos	Water safety training (out-of-water) using informational poster	1 day	Water safety knowledge 2 weeks before, 1 week after and 8 weeks after intervention
Asher et al., 1995 (47), USA	Experimental: RCT	*N* = 109 Intervention: 61, control: 48 Sex: 59 male, 50 female Age (range): 2–4 years	12 weeks of water safety training (out-of-water) and swimming skills training (in-water)	8 weeks of water safety training (out-of-water) and swimming skills training (in-water)	Intervention 12 weeks, control 8 weeks, each with 2 weekly sessions	Water safety skills Water safety behaviour Intervention: before, 8 weeks into, shortly and 12 weeks after intervention; Control: 8 weeks and shortly before, and shortly and 12 weeks after intervention
Azeredo and Stephens-Stidham, 2003 (41), USA	Experimental: non-RCT	*N* = 6,300 Intervention: 6 schools, control: 6 schools Sex: NI Age (range): 5–11 years	Water safety training (out-of-water) as part of a broader educational programme on injury prevention	No educational programme	18 or 27 weeks, 1 weekly 30- to 40-min sessions	Water safety knowledge Water safety behaviour Before and shortly after intervention
Barcala-Furelos et al., 2019 (57), Spain	Experimental: non-RCT	*N* = 26 Intervention: 14, control: 12 Sex: NI Age: 5 years	Water safety training (out-of-water) on beach and swimming pool safety and hazards using story-telling	No educational programme	1 week	Water safety knowledge Before, immediately after and 1 month after intervention
Bautista et al., 2018 (32), Spain	Experimental: non-RCT	*N* = 18 Intervention: 6, control 1: 6, control 2: 6 Sex: 16 male, 4 female Age: 4 years	Swimming skills training (in-water) with Kiflot floatation costume	1. Swimming skills training (in-water) with swimming cuffs 2. Swimming skills training (in-water) with floatation belt	7 weeks, 1 weekly 45-min session	Water safety skills Before (during first lesson) and after (during last lesson) intervention
Bradley et al., 1996 (31), Australia	Experimental: non-RCT	*N* = 33 Intervention: 17, control: 16 Sex: 19 male, 14 female Age: 6 years	Daily 30-min swimming skills training (in-water)	Weekly 30-min swimming skills training (in-water)	Intervention 2 weeks, control 10 weeks	Water safety skills Every 10 min during each lesson (highest score selected for analysis)
Brenner et al., 2009 (22), USA	Observational: case–control study	*N* = 195 61 cases, 134 controls Sex: 114 male, 81 female Age (range): 1–4 years	Risk factor: Basic swimming skills training through formal swimming lessons	No swimming skills training	NA	Drowning-related mortality
Bunker et al., 1976 (37), USA	Experimental: RCT	*N* = 36 Intervention: 18, control: 18 Sex: 18 male, 18 female Age (range): 4.5–8.5 years	Swimming skills training (in-water) with video-taped performance feedback	Swimming skills training (in-water) with auditory performance feedback	4 weeks, 1 weekly 1-h session	Water safety skills Before (during first lesson) and after (during last lesson) intervention
Cao et al., 2015 (45), China	Experimental: RCT	*N* = 1,502 Intervention: 841, control: 661 Sex: 1,174 male, 1,168 female Age (range): 8–16 years	Water safety training (out-of-water) as part of a broad, multimodal educational programme on injury prevention	Water safety training (out-of-water) using only handbook education	16 months	Water safety knowledge Water safety behaviour Before and shortly after intervention
Costa et al., 2012 (38), Portugal	Observational: retrospective cohort study	*N* = 92, but data only extracted from 32 children with 6 months of experience Intervention: 16, control: 16 Sex: NI Age (mean±SD): 4.39 ± 0.49 years	Swimming skills training in shallow water	Swimming skills training in deep water	6 months, 2 weekly 40-min sessions	Water safety skills Shortly after intervention
Erbaugh, 1986, (24), USA	Experimental: non-RCT	*N* = 94 Intervention: 30, control: 64 Sex: 63 male, 63 female Age (range): 2.5–5.5 years	Swimming skills training (in-water) through a perceptual-motor aquatic training programme	No swimming skills training	10 weeks, 2 weekly 30-min sessions	Water safety skills Before intervention, and after 4 and 8 months of intervention
Falavigna et al., 2012 (43), Brazil	Experimental: (cluster-)RCT	*N* = 1,049 Intervention: 572, control: 477 Sex: 462 male, 587 female Age: 16 years	Water safety training (out-of-water) as part of the broad “Pense Bem” injury prevention programme	No educational programme	60 min	Water safety behaviour Before, shortly after and 5 months after intervention
Frederick et al., 2000 (44), UK	Experimental: non-RCT	*N* = 1,096 Intervention: 542, control: 554 Sex: NI Age (range): 10–11 years	Water safety training (out-of-water) as part of the broad “Injury Minimization Programme for Schools”	No educational programme	5 months	Water safety knowledge Water safety behaviour Before and 5 months after intervention
Greene et al., 2002 (42), USA	Experimental: non-RCT	*N* = 1,400 Intervention: 735, control: 665 Sex: NI Age (range): 6–8 years	Water safety training (out-of-water) as part of the broad “ThinkFirst for Kids” injury prevention programme	No educational programme	6 weeks	Water safety knowledge 1 week before and 1 week after intervention
Kjendlie et al., 2009; Kjendlie and Mendritzki, 2012 (33, 63, 64), Norway	Experimental: RCT	*N* = 110 (but not all included in all study reports) Intervention: 50, control: 60 Sex: NI Age (range): 6–8 years	Swimming skills training (in-water) with “Easy Swim” personal floatation device	Swimming skills training (in-water) with minimal use of floatation aids	10 weeks, 1 weekly 40-min session	Water safety skills Immediately after (during last lesson) intervention
Kusol et al., 2020 (58), Thailand	Experimental: non-RCT	*N* = 120 Intervention: 60, control: 60 Sex: 45 male, 75 female Age (range): 7–12 years	Water safety training (out-of-water) through the “Potential Support Programme on Drowning Prevention”	No educational programme	8 weeks, 2 weekly 1-h sessions	Water safety knowledge Water safety behaviour Before and shortly after intervention
Liu et al. 2019 (56), China	Observational: case–control study	*N* = 158 79 cases, 79 controls Sex: 120 male, 56 female Age (range): 0–4 years	Risk factor: Having received water safety training (out-of-water) for unintentional drowning	No water safety training	NA	Drowning-related mortality
Mecrow et al., 2015 (49), Bangladesh	Observational: retrospective cohort study	*N* = 11,717 Intervention: 3,890, control: 3,924 Sex: NI Age (range): 6–14 years	Water safety (out-of-water) and swimming skills (in-water) training through the “SwimSafe” programme	Natural swimmers, defined as children with swimming skills not acquired through SwimSafe	NA	Water safety behaviour At the start of the swimming season and later in the season
Mecrow et al., 2015 (48), Bangladesh	Observational: retrospective cohort study	*N* = 7,046 Intervention: 3,523, control: 3,438 Sex: 3,608 male, 3,438 female Age (range): 5–14 years	Water safety (out-of-water) and swimming skills (in-water) training through the “SwimSafe” programme	Natural swimmers, defined as children with swimming skills not acquired through SwimSafe	NA	Water safety behaviour At the start of the swimming season and later in the season
Misimi et al., 2022 (35), Kosovo	Experimental: RCT	*N* = 36, with fear of water Intervention: 17, control: 19 Sex: NI Age (range): 10–11 years	Swimming skills training (in-water) with goggles and snorkel	Swimming skills training (in-water) without goggles and snorkel	4 weeks, 5 weekly 45-min sessions	Water safety skills 2 days before and shortly after intervention
Misimi et al., 2023 (36), Kosovo	Experimental: RCT	*N* = 34, without fear of water Intervention: 16, control: 18 Sex: NI Age (range): 10–11 years	Swimming skills training (in-water) with goggles and snorkel	Swimming skills training (in-water) without goggles and snorkel	4 weeks, 5 weekly 45-min sessions	Water safety skills 2 days before and shortly after intervention
Moreno-Murcia et al., 2016 (30), Spain	Experimental: non-RCT	*N* = 16 Intervention: 8, control: 8 Sex: 7 male, 9 female Age (range): 3–5 years	Swimming skills training (in-water) using motility stories	Swimming skills training (in-water) using a traditional approach without motility stories	6 weeks, 2 weekly 40-min sessions	Water safety skills Before and shortly after intervention
Moura et al., 2021 (26), Portugal	Experimental: RCT	*N* = 31 Intervention: 17, control: 14 Sex: 7 male, 9 female Age (range): 7–9 years	Swimming skills training (in-water) focussing on basic aquatic skill development	Swimming skills training (in-water) focussing on formal swimming technique	6 weeks, 1 weekly 50-min session	Water safety skills Before and shortly after intervention
Papadimitriou and Loupos, 2021 (27), Greece	Experimental: RCT	*N* = 23 Intervention: 12, control: 11 Sex: 13 male, 10 female Age (range): 8–10 years	Swimming skills training (in-water) through the “Tec Pa” swimming programme	Swimming skills training (in-water) through standard exercises with high repetition	8 weeks, 3 weekly 45-min sessions	Water safety skills Before intervention, mid-intervention and shortly after intervention
Parker et al., 1999 (34), Australia	Experimental: non-RCT	*N* = 19 Intervention: 10, control: 9 Sex: 12 male, 7 female Age (range): 6–7 years	Swimming skills training (in-water) using multiple buoyancy and propulsion aids	Swimming skills training (in-water) using only a kickboard	2 weeks, 2 weekly 40-min sessions	Water safety skills Water safety behaviour Before (during first lesson) and after (during last lesson) intervention
Pilgaard et al., 2020 (25), Sweden	Observational: retrospective cohort study	*N* = 3,468 Intervention: 1,695, control: 1,773 Sex: 50.8% male, 49.2% female Age (range): 10–11 years	Swimming skills training (in-water) through the “School Swim programme”	No school-regulated educational programme; swimming education differed across schools	3 weeks, 5 weekly 45-min sessions	Water safety skills 2 years after intervention
Pratt et al., 2023 (28), UK	Experimental: (cluster-)RCT	*N* = 107 Intervention: 53, control: 54 Sex: 52 male, 55 female Age (mean±SD): 7.8 ± 0.63 years	Swimming skills training (in-water) through a progressive aquatic motor competence programme	Swimming skills training (in-water) through traditional swimming lessons	6 weeks, 1 weekly 30- or 45-min session	Water safety skills Before and shortly after intervention
Rahman et al., 2012 (46), Rahman et al., 2020 (65), Bangladesh	Observational: retrospective cohort study	*N* = 160,470 Intervention: 57,834, control: 102,636 Sex: 53.6% male and 46.6% female (intervention), 49.3% male and 50.7% female (control) Age (range): 4–12 years	Swimming skills training (in-water) and water safety training (out-of-water) through the “SwimSafe” programme	No educational programme	3 weeks	Drowning-related mortality Cost-effectiveness
Rocha et al., 2018 (39), Portugal	Experimental: non-RCT	*N* = 21 Intervention: 10, control: 11 Sex: NI Age (mean±SD): 4.70 ± 0.51 years	Swimming skills training in shallow water	Swimming skills training in deep water	6 months, 2 weekly 45-min sessions	Water safety skills Before (during first session) and shortly after intervention
Shen et al., 2016 (21), USA	Experimental: RCT	*N* = 280 Intervention: 137, control: 143 Sex: 137 male, 143 female Age (mean±SD): 10.03 ± 0.83 years	Water safety training (out-of-water) through testimonial videos of drowning experiences	No water safety training; testimonial videos on dog bite prevention	36 min	Water safety knowledge Water safety behaviour 1 week after intervention
Simon-Piqueras et al., 2022 (29), Spain	Experimental: RCT	*N* = 17 Intervention: 8, control: 9 Sex: 7 male, 10 female Age (range): 4–5 years	Swimming skills training (in-water) through aquatic motor games and repetitive aquatic motor competence exercises	Swimming skills training through repetitive aquatic motor competence exercises only	6 weeks, 2 weekly 1-h sessions	Water safety skills Before and shortly after intervention
Terzidis et al., 2007 (59), Greece	Experimental: non-RCT	*N* = 1,400 Intervention: 641, control: 759 Sex: 681 male, 719 female Age (range): 5–15 years	Water safety training (out-of-water) through a classroom programme including presentations, discussions and drama plays	No educational programme	1 day	Water safety knowledge Water safety behaviour 1 month after intervention
Yang et al., 2007 (23), China	Observational: case–control studies	*N* = 379113 cases, 266 controls Sex: 240 male, 159 female Age (range): 1–14 years	Risk factor: Having received basic swimming skills training through formal swimming lessons	No swimming skills training	NA	Drowning-related mortality

RCT, randomized controlled trial; NA, not applicable; NI, no information.

a Reported number of participants is the number after drop-out.

In eighteen studies, the intervention comprised an educational programme delivering basic swimming skills (in-water). Of these, four studies compared a swimming skills intervention to no swim training, five compared different pedagogical approaches, one study compared different training frequencies, one study compared different modes of delivery for swimming skills training, five studies addressed the use of didactic materials, and two studies focused on the setting of swim training. Isolated water safety skills intervention programmes (out-of-water) and water safety training as part of a broader educational programme on injury prevention were explored in six and five studies, respectively. In four studies, the intervention comprised a combination of basic swimming skills (with in-water training) and water safety skills (with out-of-water training).

### 3.3 Risk of bias and certainty of the evidence

**Figure 2:**
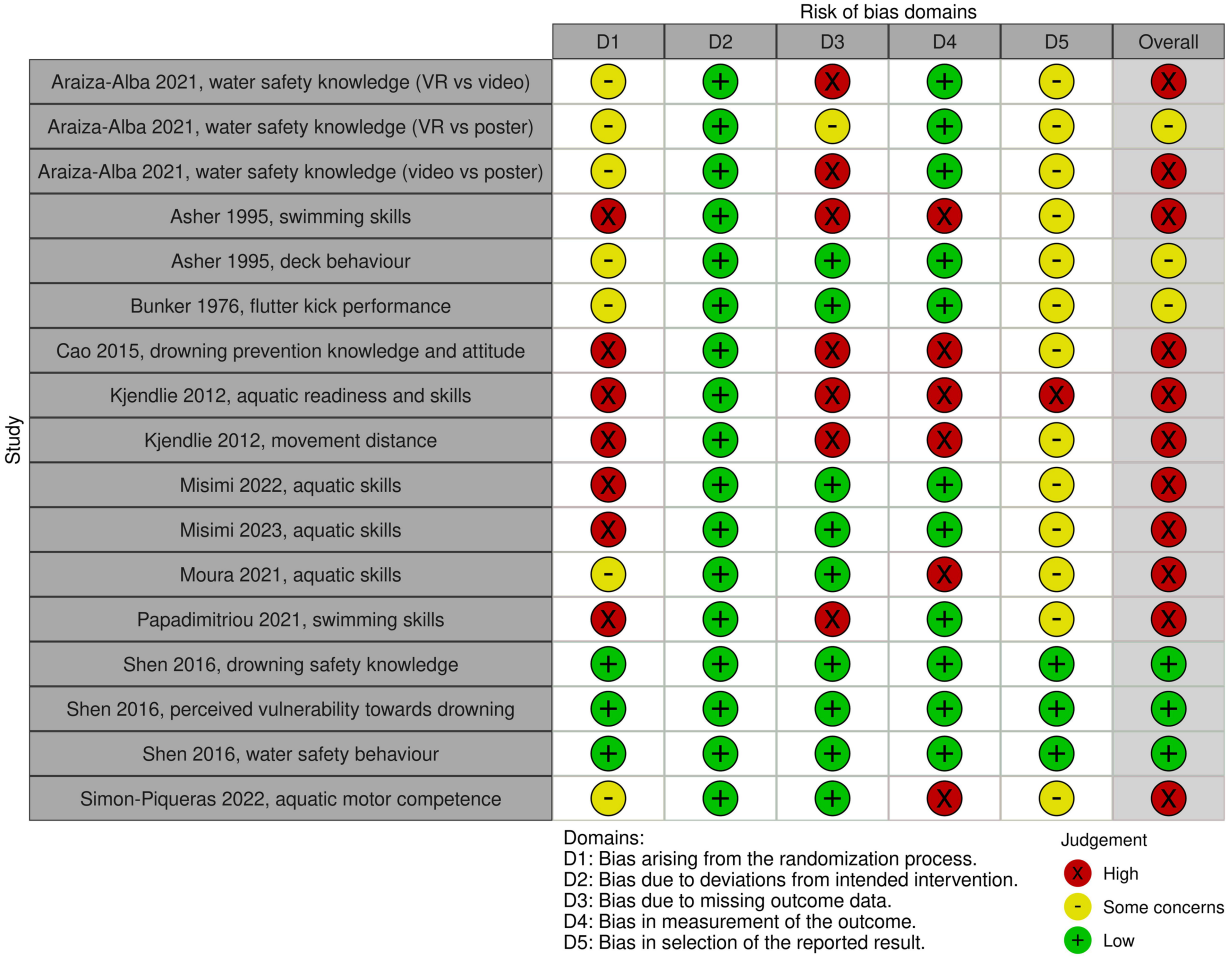
Risk of bias for each outcome of interest of parallel-randomized controlled trials, as assessed using the Cochrane Risk of Bias-2 too.

**Figure 3:**
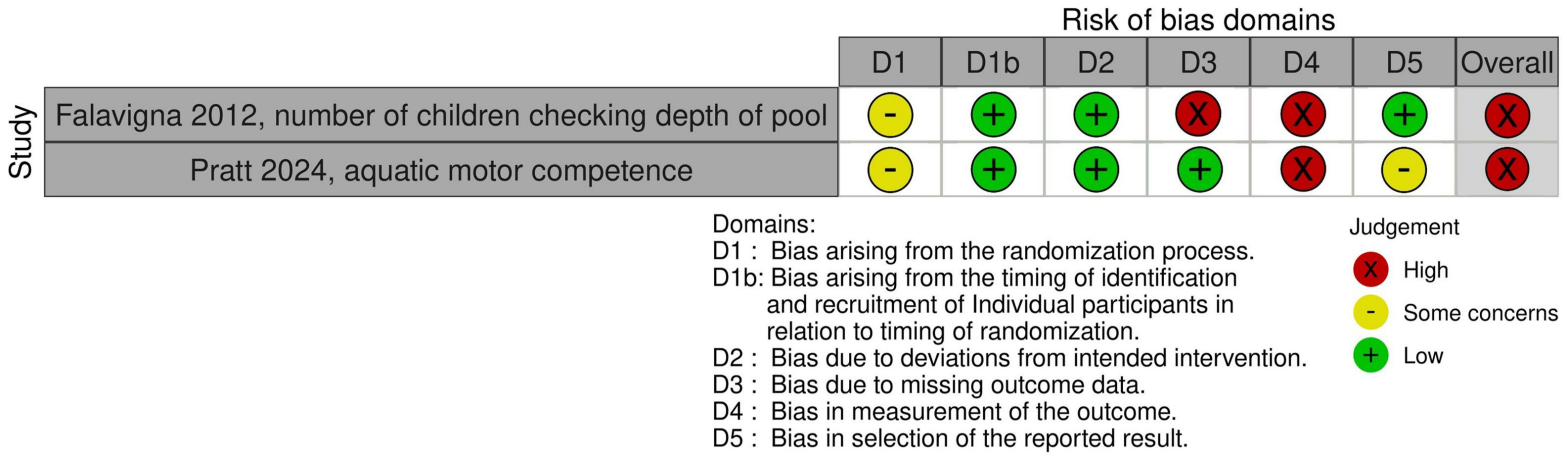
Risk of bias for each outcome of interest of cluster-randomized controlled trials, as assessed using the Cochrane Risk of Bias-2 tool.

The risk of bias for the outcomes of interest in the individual parallel and cluster-randomized controlled trials is presented in Figures 2 and 3, respectively. Overall risk of bias was high for 13 out of the 19 outcomes measured across the included RCTs. High risk of bias arose mainly from insufficient details on the methods used for randomization and/or allocation concealment, lack of blinding of outcome assessors, lack of a pre-specified analysis plan and missing outcome data potentially being attributable to the true value of the data. For 3 out of the 19 outcomes, a moderate risk of bias was shown and 1 study (21) had an overall low risk of bias.

**Figure 4:**
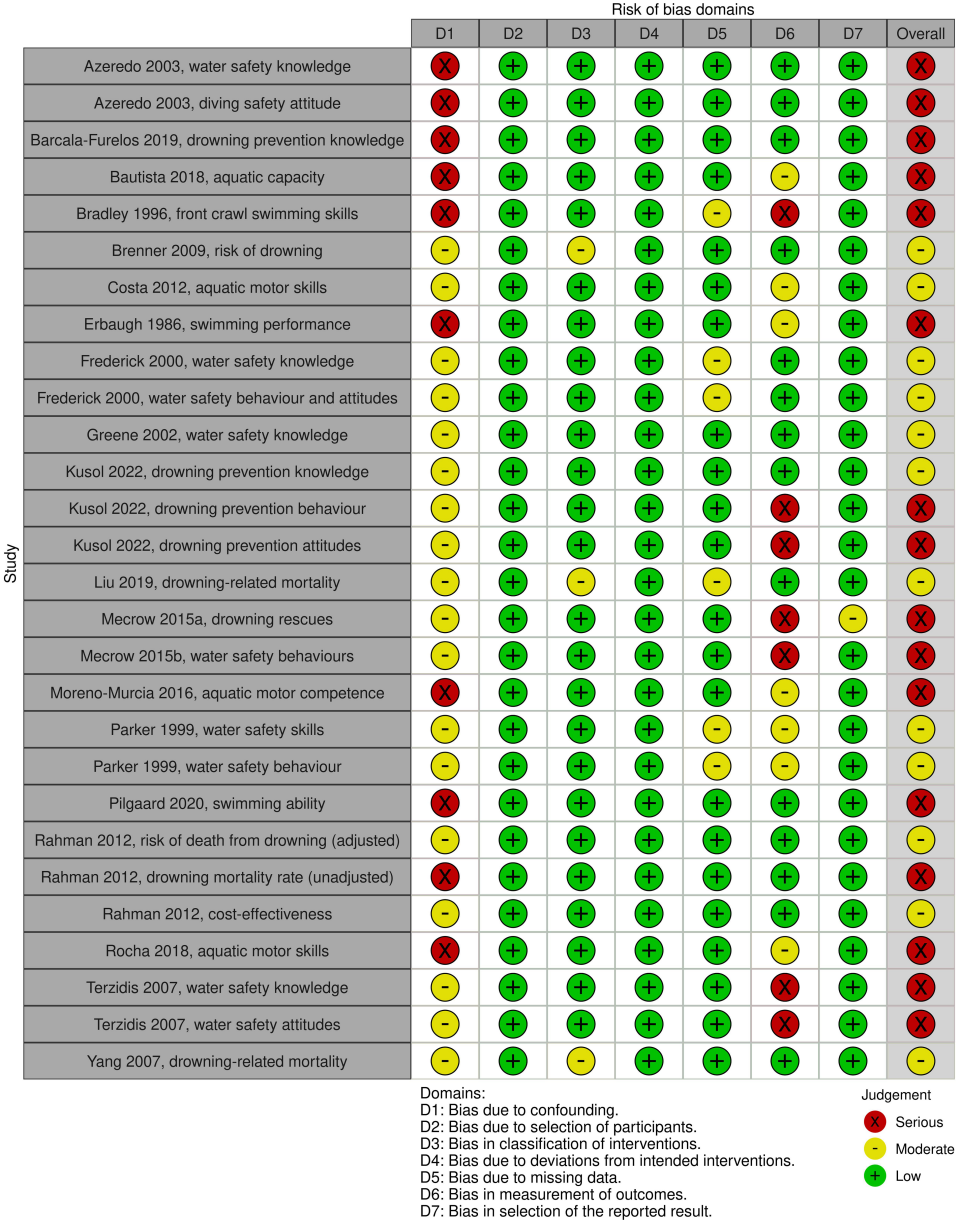
Risk of bias for each outcome of interest of non-randomized experimental studies and observational studies, as assessed using the ROBINS-I tool.

For the outcomes of the included non-randomized experimental and observational studies, risk of bias is visualized in Figure 4. Overall risk of bias was high for 16 out of the 28 outcomes studied in the included non-RCTs and observational studies. For the remaining outcomes, moderate concerns were raised. None of the studies adequately controlled for confounding factors, such as demographic variables or the pre-intervention skills level. Also, the use of subjective outcome measures, along with a lack of blinding of the outcome assessors, resulted in moderate to serious risk of bias in the measurement of the outcome in the majority of studies.

Certainty of evidence was rated low or very low for nearly all outcomes. Overall, serious limitations in the design of the included studies were identified, leading to the certainty of the evidence being downgraded by one or two levels for nearly all outcomes [with the exception of (21)]. In addition, several outcomes were downgraded (by one or two levels) due to imprecision, stemming from a lack of data, limited sample size, low number of events and/or a wide confidence interval around the effect estimate. There was no indication of indirectness or publication bias for any of the studied outcomes. Also, none of the outcomes were downgraded for heterogeneity.

### 3.4 Synthesis of findings

In the following paragraphs, the findings concerning the effectiveness of basic swimming skills and water safety interventions are narratively summarized. For detailed synthesis of findings tables for each individual outcome along with the grading of the certainty of evidence, see Appendix 4.

#### 3.4.1 Basic swimming skills (in-water) training

Eighteen studies, including eight RCTs, six non-RCTs, two cohort studies and two case–control studies, implemented an in-water basic swimming skills intervention. Results of these studies and certainty of the evidence are summarized in Table 2.

**Table 2:** Overview of studies comparing a basic swimming skills (in-water) training to no or another basic swimming skills training, with an indication of results and the certainty of evidence, assessed based on the GRADE approach.

Intervention vs control		Drowning-related mortality	Water safety skills	Water safety knowledge	Water safety behaviour
Swimming skills training vs no training					
Swimming skills training vs. no swimming skills training	+	Brenner et al., 2009 (22) (*n* = 1) ^Low^ Yang et al., 2007 (23) (*n* = 1) ^Low^	Erbaugh, 1986 (24), (*n* = 6) ^Very low^	/	/
	~	Yang et al., 2007 (23) (*n* = 1) ^Very low^	Pilgaard et al., 2020 (25) (*n* = 1) ^Very low^		
Pedagogical approach to basic swimming skills (in-water) training					
Swimming skills training using motility stories vs. traditional swimming skills training	~	/	Moreno-Murcia et al., 2016 (30) (*n* = 1) ^Very low^	/	/
Basic swimming skills training vs. formal swimming skills training	+	/	Moura et al., 2021 (26) (*n* = 7) ^Very low^	/	/
	~		Moura et al., 2021 (26) (*n* = 10) ^Very low^		
Aquatic motor competence training vs. traditional swimming skills training	+	/	Pratt et al., 2023 (28) (*n* = 1) ^Very low^	/	/
	~		Pratt et al., 2023 (28) (*n* = 2) ^Very low^		
Tec Pa swimming skills training programme vs. traditional swimming skills training	+	/	Papadimitriou and Loupous, 2021 (27) (*n* = 3) ^Very low^	/	/
	~		Papadimitriou and Loupous, 2021 (27) (*n* = 4) ^Very low^		
Swimming skills training using aquatic motor games vs. traditional swimming skills training	+	/	Simon-Piqueras et al., 2022 (29) (*n* = 1) ^Very low^	/	/
	~		Simon-Piqueras et al., 2022 (29) (*n* = 1) ^Very low^		
Frequency of basic swimming skills (in-water) training					
Daily swimming skills training vs. weekly swimming skills training	~	/	Bradley et al., 1996 (31) (*n* = 1) ^Very low^	/	/
Didactic material use for basic swimming skills (in-water) training					
Swimming skills training with buoyancy aids vs. swimming skills training without buoyancy aids	+	/	/	/	Kjendlie and Mendritzki, 2012 (33) (*n* = 1) ^Very low^
	~		Kjendlie and Mendritzki, 2012 (33) (*n* = 12) ^Very low^ Parker et al., 1999 (34) (*n* = 3) ^Very low^ Bautista et al., 2018 (32) (*n* = 1) ^Very low^		Kjendlie and Mendritzki, 2012 (33) (*n* = 2) ^Very low^ Parker et al., 1999 (34) (*n* = 1) ^Very low^
Swimming skills training with goggles and snorkel vs. swimming skills training without goggles and snorkel	+	/	Misimi et al., 2022 (35) (*n* = 3) ^Very low^ Misimi et al., 2023 (36) (*n* = 1) ^Very low^	/	/
	~		Misimi et al., 2022 (35) (*n* = 7) ^Very low^ Misimi et al., 2023 (36) (*n* = 8) ^Very low^		
	−		Misimi et al., 2022 (35) (*n* = 1) ^Very low^ Misimi et al., 2023 (36) (*n* = 2) ^Very low^		
Mode of delivery of basic swimming skills (in-water) training					
Swimming skills training with video-taped feedback vs. swimming skills training with auditory feedback	+	/	Bunker et al., 1976 (37) (*n* = 1) ^Low^	/	/
	~		Bunker et al., 1976 (37) (*n* = 1) ^Low^		
Setting of basic swimming skills (in-water) training					
Swimming skills training in shallow water vs. swimming skills training in deep water	+	/	Costa et al., 2012 (38) (*n* = 14) ^Low^ Rocha et al., 2018 (39) (*n* = 6) ^Very low^	/	/
	~		Costa et al., 2012 (38) (*n* = 3) ^Low^ Rocha et al., 2018 (39) (*n* = 11) ^Very low^		

+ results in favour of the intervention; ~ results neither in favour of the intervention nor control; − results in favour of the control; n indicates the number of outcomes. Certainty of evidence based on GRADE is indicated in superscript.

Four studies investigated the effect of swimming skills training compared to no training on either drowning-related mortality or water safety skills. Two case–control studies revealed a statistically significant reduction in drowning-related mortality in children aged 0 to 4 years who received formal training on swimming skills (low certainty evidence (22, 23)). These results could not be replicated in a group of 5 to 14 year-old children (very low certainty evidence (23)). Comparing a perceptual-motor aquatic training programme to no swim training, a statistically significant enhancement in six swimming skills domains was observed in children under the age of 6 years (very low certainty evidence (24)). Pilgaard et al. (25) could not demonstrate an increase in elementary school children’s self-reported ability to swim 200 metres, 2 years after the implementation of the School Swim programme in Sweden, compared to a pre-intervention cohort.

In five studies with a (non-)randomized experimental design, the effectiveness of an experiential or playful pedagogical approach to swimming skills training, compared to traditional or formal swim training, was investigated. A statistically significant increase in water safety skills, delivering basic swimming skills training (6/17 outcomes), aquatic motor competence training (1/3 outcomes), the Tec Pa training programme (3/7 outcomes) and swimming skills training using aquatic motor games (1/2 outcomes), compared to traditional or formal swimming skills training in children under the age of 6 years or elementary school children, was demonstrated (26–29). No evidence of swim training using motility stories being superior to traditional swim training to improve aquatic motor competence in 3 to 5 year old children was found (30). All evidence is of very low certainty.

Frequency of basic swimming skills training was targeted in one non-RCT (31). Based on very low certainty evidence, the authors could not demonstrate a difference in front crawl swimming performance following daily compared to weekly swim training sessions in 6 year olds.

The effectiveness of didactic material use for swimming skills training was studied in five experimental studies. Three targeted the use of buoyancy aids, while two focused on the use of goggles and snorkels. A statistically significant increase in water safety skills, using buoyancy aids compared to no buoyancy aids in teaching swimming skills to children aged 4 to 8 years, could not be demonstrated in any of the 16 outcomes studied (32–34). Likewise, for the majority of outcomes on water safety behaviour (3/4 outcomes), no significant difference was found between children trained with or without a buoyancy aid (33, 34). Kjendlie et al. (33) did register an increased number of surface dives during free play in children who had received swimming skills training without a floatation aid, compared to children who were trained with a floatation aid. With respect to the use of goggles and snorkels, inconsistent evidence was found. On the one hand, enhanced water safety skills were observed in both children with a high fear of water (3/11 outcomes, (35)) and children with no fear of water (1/11 outcomes, (36)) who had received swimming skills training with goggles and snorkels, compared to children who had received training without these aids. Specifically, a significantly larger improvement from pre- to postintervention was shown for the water safety test, back gliding test and prone swim test in children with a high fear of water, and the prone swim test alone in children with a low fear of water. On the other hand, for some other water safety skills (1/11 and 2/11 outcomes in children with high and low fear of water, respectively), children who had used goggles and snorkels during training demonstrated a significantly lower pre- to postintervention improvement. Specifically, this was observed for the blowing bubbles test in both the high and low fear group, and the breathing during prone swimming test in the low fear group. For the remainder of the outcomes, a significant between-group difference could not be demonstrated. All evidence relating to the use of didactic materials is of very low certainty.

The use of video-taped compared to auditory feedback in delivering swimming skills training was compared in one RCT (37). Low certainty evidence showed a statistically significant increase in flutter kick performance using video-taped feedback in children aged 6.5 to 8.5 years, but not in a group of children under the age of 6 years.

Lastly, two studies (one RCT and one retrospective cohort study) examined the effectiveness of swimming skills training in shallow compared to deep water. Teaching basic swimming skills in shallow water in children under the age of 6 years resulted in a statistically significant improvement in the majority of the aquatic skills studied (20/34 outcomes, low to very low certainty evidence). For 14 outcomes, a significant difference in water safety skills dependent on water depth during swimming skills training could not be demonstrated (38, 39).

#### 3.4.2 Water safety (out-of-water) training

Results on the effectiveness of water safety (out-of-water) training, along with the certainty of the evidence, are summarized in Table 3. Five studies (one RCT, three non-RCTs, 1 case–control study) were identified, providing a total of 19 effect estimates on the effectiveness of water safety training without an in-water component compared to no training. Water safety training was shown to significantly decrease drowning-related mortality (1/1 outcome, low certainty evidence), increase water safety knowledge (9/11 outcomes, high to very low certainty evidence) and increase water safety behaviour (5/7 outcomes, high to very low certainty evidence) in children under the age of 6 years and elementary school children. For two water safety knowledge and two water safety behaviour outcomes, a significant difference comparing water safety training to no training could not be demonstrated (very low certainty evidence).

**Table 3:** Overview of studies comparing a water safety skills (out-of-water) training to no or another water safety skills training, with an indication of results and the certainty of evidence, assessed based on the GRADE approach.

Intervention vs control		Drowning-related mortality	Water safety skills	Water safety knowledge	Water safety behaviour
Water safety training vs no training					
Water safety training vs. no water safety training	+	Liu et al., 2019 (56) (*n* = 1) ^Low^	/	Shen et al., 2016 (21) (*n* = 1) ^High^ Kusol et al., 2020 (58) (*n* = 1) ^Moderate^ Terzidis et al., 2007 (59) (*n* = 1) ^Very low^ Barcala-Furelos et al., 2019 (57) (*n* = 6) ^Very low^	Shen et al., 2016 (21) (*n* = 2) ^High^ Kusol et al., 2020 (58) (*n* = 2) ^Low^ Terzidis et al., 2007 (59) (*n* = 1) ^Very low^
	~	/		Terzidis et al., 2007 (59) (*n* = 2) ^Very low^	Terzidis et al., 2007 (59) (*n* = 2) ^Very low^
Mode of delivery of water safety (out-of-water) training					
Water safety training using virtual reality vs. water safety training using traditional methods	~	/	/	Araiza-Alba et al., 2021 (40) (*n* = 1) ^Very low^	/

+ results in favour of the intervention; ~ results neither in favour of the intervention nor control; − results in favour of the control; n indicates the number of outcomes. Certainty of evidence based on GRADE is indicated in superscript.

One RCT compared the effectiveness of water safety training using virtual reality to traditional training methods in children aged 10–12 years. A statistically significant difference in water safety knowledge between training methods could not be demonstrated (40).

#### 3.4.3 Water safety training as part of a broad injury-prevention programme

Table 4 provides a synthesis of the results and certainty of evidence of studies targeting water safety training as part of a broad injury prevention programme. In four experimental studies (one RCT, 3 non-randomized trials), the effectiveness of a broad injury prevention programme comprising a component on water safety (e.g., Injury Minimization Programme for Schools, ThinkFirst, PenseBem), was explored. Injury prevention programmes, delivered to elementary school children, were shown to significantly increase knowledge on water safety (5/6 outcomes, low to very low certainty evidence (41, 42)). In contrast, with respect to water safety behaviour, inconsistent results were obtained. Increased water safety behaviour in children participating in an intervention programme was observed for four outcomes (low to very low certainty evidence (41–43)), that is, the attitude on diving safety, the ability to identify the risk of a toddler falling in water, the ability to decide to stop playing near water, and the number of children checking the depth of a swimming pool. In contrast, for three other outcomes of interest (i.e., the ability to identify general water danger, identify the risk of playing with a ball near water, and decide not to play near water), children who did not receive education on injury prevention and water safety exhibited safer behaviour (low certainty evidence (42)). For one outcome on water safety knowledge (44) and one outcome on water safety behaviour (43), a statistically significant between-group difference could not be demonstrated.

**Table 4:** Overview of studies comparing a water safety (out-of-water) training as part of a broad educational programme on injury prevention to no or another training, with an indication of results and the certainty of evidence, assessed based on the GRADE approach.

Intervention vs control		Drowning-related mortality	Water safety skills	Water safety knowledge	Water safety behaviour
Educational programme with water safety training vs no training					
Water safety training as part of a broad educational programme vs. no water safety training	+	/	/	Azeredo and Stephens-Stidham, 2003 (41) (*n* = 2) ^Very low^ Greene et al., 2002 (42) (*n* = 3) ^Low^	Azeredo and Stephens-Stidham, 2003 (41) (*n* = 1) ^Very low^ Greene et al., 2002 (42) (*n* = 2) ^Low^ Falavigna 2012 (*n* = 1) ^Low^
	~			Frederick et al., 2000 (44) (*n* = 1) ^Low^	Falavigna et al., 2012 (43) (*n* = 1) ^Low^
	−			/	Greene et al., 2002 (42) (*n* = 3) ^Low^
Mode of delivery of water safety (out-of-water) training					
Water safety training as part of a multimodal education programme vs. water safety training using traditional handbook education	~	/	/	Cao et al., 2015 (45) (*n* = 1) ^Low^	/

+ results in favour of the intervention; ~ results neither in favour of the intervention nor control; − results in favour of the control; n indicates the number of outcomes. Certainty of evidence based on GRADE is indicated in superscript.

Based on one RCT comparing injury prevention education through a multimodal programme to traditional handbook education in elementary school and adolescent children, a difference in water safety knowledge dependent on the mode of delivery of water safety training could not be observed (45). This evidence was of low certainty.

#### 3.4.4 Combined basic swimming skills (in-water) and water safety skills (out-of-water) training

A combined basic swimming skills (in-water) and water safety (out-of-water) training programme (Table 5) was delivered in four studies: three retrospective cohort studies on the SwimSafe programme, implemented in a LMIC, and one RCT that was carried out in a HIC. Evidence of moderate certainty showed a statistically significant decrease in the risk of death from drowning in children aged 4–12 years, comparing the implementation of the SwimSafe programme to no programme. For the drowning mortality rate assessed by year of age (very low certainty evidence), a significant difference could not be demonstrated (46). In addition, following a training programme on swimming skills and water safety, enhanced water safety skills were noted for nearly all outcomes of interest (5/6 outcomes, very low certainty evidence), with the exception of the ability to jump and swim after an 8-week training programme (very low certainty evidence (47)). In contrast, for almost all outcomes relating to water safety behaviour (17/18 outcomes, low to very low certainty evidence), a statistically significant difference could not be observed between children enrolled in a combined swimming and water safety training programme, compared to children who had not participated in such a programme (47) or compared to natural swimmers (48, 49). However, the number of drowning rescues ever performed by children who had participated in a swimming skills and water safety programme was significantly higher compared to non-swimming elementary school and adolescent children (very low certainty evidence (49)). Swimsafe was shown to be a highly cost- effective programme, at an average cost per death averted and cost per disability-adjusted life year (DALY) averted of $3,009 and $85, respectively.

**Table 5:** Overview of studies comparing a combined basic swimming skills (in-water) and water safety skills (out-of-water) training to no or another training, with an indication of results and the certainty of evidence, assessed based on the GRADE approach.

Intervention vs control		Drowning-related mortality	Water safety skills	Water safety knowledge	Water safety behaviour
Swimming skills and water safety training vs no training					
Swimming skills and water safety training vs. no swimming skills or water safety training	+	Rahman et al., 2012 (46) (*n* = 1) ^Moderate^	Asher et al., 1995 (47) (*n* = 5) ^Very low^	/	Mecrow et al., 2015 (49) (*n* = 1) ^Very low^
	~	Rahman et al. 2012 (46) (*n* = 9) ^Very low^	Asher et al. 1995 (47) (*n* = 1) ^Very low^	/	Asher et al. 1995 (47) (*n* = 2) ^Very low^ Mecrow et al. 2015 (49) (*n* = 9) ^Very low^ Mecrow et al. 2015 (48) (*n* = 3) ^Low^ Mecrow et al. 2015 (48) (*n* = 3) ^Very low^
Duration of combined basic swimming skills (in-water) and water safety (out-of-water) training					
12-week swimming and water safety skills training vs. 8-week swimming and water safety skills training	~	/	Asher et al. 1995 (47) (*n* = 4) ^Very low^	/	Asher et al. 1995 (47) (*n* = 2) ^Low^

+ results in favour of the intervention; ~ results neither in favour of the intervention nor control; − results in favour of the control; *n* indicates the number of outcomes. Certainty of evidence based on GRADE is indicated in superscript.

## 4 Discussion

This systematic review aimed to update the existing body of evidence on the effect of basic swimming skills and/or water safety training on drowning-related mortality and morbidity, and water safety skills, knowledge and behaviour, synthesized in the 2021 WHO Guideline on Drowning Prevention (14). In the current update, the review was supplemented with 12 studies, most of which explored a training programme for swimming skills, resulting in the inclusion of 33 studies in total. All newly identified studies were relevant to the secondary aim of this systematic review, i.e., identifying determining characteristics for basic swimming skills and water safety programme effectiveness. None of these studies addressed the primary objective relating to drowning mortality and morbidity.

### 4.1 Basic swimming skills training

Our original systematic review, conducted in 2019, revealed that education in basic swimming skills, compared to providing no training, may reduce drowning-related mortality in children aged 1–4 years (22, 23), and may improve performance in a variety of aquatic competences in preschool children (24). The current update identified nine new studies that addressed the effectiveness of different implementation approaches for basic swimming skills training.

With regard to the pedagogical approach used, programmes adopting a non-traditional approach to swim training, prioritizing basic aquatic skills or aquatic motor competence rather than formal swimming techniques, may result in a considerably higher gain in a number of aquatic competences. This observation was made in four out of the five experimental studies comparing an experiential or playful approach to a traditional swim training, in both elementary school (26–28) and preschool children (29). For a motility story-based programme targeted at children between the ages of 3 to 5 years, no such effect was observed. The common denominator amongst these non-traditional programmes is their primary focus on familiarization with an aquatic environment and development of motor awareness and fundamental aquatic competences, before targeting formal swimming techniques. It has been argued that acquiring basic competences and gaining confidence in an aquatic environment form a prerequisite for learning complex swimming skills (50), reflecting the more general principle that the acquisition of high-complexity motor skills builds upon the consolidation of low-complexity skills (51). In line with this notion, children enrolled in non-traditional training programmes not only demonstrated enhanced improvement in basic aquatic competency outcomes (e.g., buoyancy, deep water immersion), but also in several swimming techniques (e.g., back- and breaststroke swimming technique), compared to children who received training centred on traditional techniques (26–29).

The impact of using didactic materials during swimming skills training is less straightforward. There was insufficient evidence to show that using flotation equipment results in enhanced water safety skills and behaviour (32–34). Using goggles and snorkels may, on the one hand, facilitate the acquisition of some aquatic competences in elementary school children with and without fear of water prior to enrolment in a training programme. On the other hand, the use of this equipment may also interfere with the acquisition of skills that require one to expire in water (i.e., blowing bubbles, breathing during prone swimming) (35, 36). Since the latter skills are crucial for developing a correct breathing technique during swimming, the present findings suggest that relying heavily on these materials during swim practice may be counterproductive.

Evidence from a single RCT suggests that the use of video-taped feedback during swim training may aid children over the age of 6 years in learning technical swimming skills, in line with the general supportive effect of video-taped feedback that has been repeatedly demonstrated for motor learning (52–54). Importantly, there was insufficient evidence to demonstrate a similar effect in children under the age of 6 years (37), raising the question as to whether preschool children demonstrate sufficient cognitive and perceptual abilities to learn from video images.

Most of the studies that delivered a swimming skills intervention targeted children of elementary school age. According to the WHO, however, in particular younger children account for a disproportionally large portion of drowning fatalities (1). In addition, Langendorfer et al. (55) argued that, in terms of reducing the risk of drowning, children can benefit from aquatic training from as early as the age of 1 year. The current body of evidence demonstrates a beneficial effect of providing basic swimming skills and water safety education in early childhood. However, this review offers limited guidance on what strategies are most effective for delivering basic swim training in preschool children in terms of pedagogical approach (30), the use of buoyancy aids (34) and training duration (47). Two studies did demonstrate that swim training in shallow water, compared to deep water, may significantly enhance performance on a number of aquatic competences in preschoolers (38, 39). This observation largely corresponds to the overall conclusion of the systematic review by Mekkaoui et al. (13), showing a lack of agreement on which instructional methods yield the most favourable change in aquatic literacy in children between the ages of 4 and 6 years.

### 4.2 Water safety (out-of-water) training

Evidence stemming from a single observational study suggests that training children under the age of 4 years in water safety may reduce drowning-related mortality (56). In addition, providing children and adolescents with water safety training, either isolated or as part of a broad educational program on injury prevention, is effective in increasing knowledge on aquatic safety and reducing unsafe behaviour in and around water (21, 41–44, 57–59). With the current update introducing limited new evidence from only one study (58), albeit of moderate certainty, these conclusions remain largely stable relative to our 2019 review.

Water safety programmes ranged from a one-hour testimonial-based intervention to multi-component programmes delivered over the course of weeks or months. The consistent observation of the beneficial effect of educating children in water safety across all implemented interventions suggests that even simple, short interventions may considerably impact safety in elementary school children. Nevertheless, reliable evidence from direct between-intervention comparisons, allowing us to identify the most (cost-)effective approach in terms of programme content, duration and teaching methods, is lacking.

### 4.3 Combined swimming and water safety training

Four studies assessed the effectiveness of combined in-water swimming training and out-of-water safety education. One RCT showed that providing preschool children with training in swimming skills and water safety may improve swimming abilities, but there was insufficient evidence to suggest safer behaviour in and around water (47). The remaining three studies reported on the roll-out of the SwimSafe programme, combining training in basic swimming skills, water safety and safe rescue techniques in children aged 4 to 14 years, as part of a larger injury prevention program in rural Bangladesh (46, 48, 49). The SwimSafe programme is likely effective in reducing the overall risk of death from drowning. In addition, children enrolled in the programme, compared to non-swimmers, may perform a greater number of drowning rescues. On the one hand, this finding suggests that SwimSafe-trained children may show enhanced competence and confidence in an aquatic environment, leading them to perform more (successful) drowning rescues compared to their non-trained peers. This interpretation is further supported by a report on rescuer-drowning in Turkey, showing that the promotion of in-water swimming and safe rescue skills may reduce the risk of multiple drowning incidents (i.e., incidents where two or more individuals drown at the same time) (60). On the other hand, the incidence of adverse events as a consequence of SwimSafe, in particular the number of rescuer-drownings, have not been reported, leaving it unclear whether the overall reduction in submersion fatalities may come at a cost of increased rescue-related mortality.

The SwimSafe programme proves to be a highly cost-effective educational intervention, both when implemented as an isolated intervention or complemented with the Anchal daycare programme for preschool children (46). At an average cost/DALY averted of $85, the intervention could be particularly suitable for low-resource settings.

### 4.4 Limitations and directions for future research

This systematic review provides an elaborate synthesis of the evidence on swimming skills and water safety education as a drowning prevention strategy in children under the age of 18 years and supports the conclusion of the WHO Guideline on drowning prevention on the overall effectiveness of these interventions. In addition, this update provides guidance on the most effective implementation approaches to basic swimming skills and water safety training. In doing so, this review can serve as a valuable resource for informing educators and swimming instructors in the design and implementation of aquatic training programs. Nevertheless, relying solely on the education of children in basic swimming skills is likely insufficient to achieve sustainable safe behaviour in and around water. Coordinated engagement of diverse stakeholders (e.g., parents, teachers, schools, pedagogists, policy-makers) are needed to create a lasting, widely supported impact (10). Also, it must be emphasized that educating children in aquatic safety and swimming skills constitutes only one component of the multi-domain and -sectoral action that is required to significantly reduce the global burden of drowning (10).

Also, some limitations associated with this review and the available evidence should be addressed. The evidence synthesized in this review is characterized by a substantial risk of bias in the study design, resulting in an overall low to very low certainty evidence base. Adding to this is the large heterogeneity in the studied populations (in terms of age), implemented interventions and measured outcomes, precluding us from performing meta-analyses and attaining high-precision evidence. In addition, the impact of basic swimming skills and water safety training on the primary outcomes (i.e., drowning mortality and morbidity) was reported in only a limited number of studies. While a considerable amount of evidence points towards enhanced water safety knowledge, skills and behaviour after training, these outcomes do not necessarily translate to a reduced risk of death from drowning. The scarcity of reliable drowning data calls for robust drowning monitoring systems and comprehensive reporting on government-implemented drowning prevention programmes targeting large communities. These systemic changes could aid in generating high-certainty evidence by addressing the general underestimation of drowning mortality rates (61, 62) and the limited precision of the current body of evidence. Alternatively, we must also critically reflect on how reliably drowning-related outcomes can capture the intended goal of water safety. That is, evidence on drowning mortality typically stems from case–control studies and retrospective cohort studies, as was also the case in the current review (22, 23, 46, 56). While these types of investigations can provide an indication of the association between water competence and the risk of drowning, observational designs lack the methodological rigour to establish causality. Confounding factors, such as the availability of safe swimming areas and the presence of supervision, may skew drowning mortality and morbidity results. Considering the challenges in conducting solid RCTs to assess the impact of basic swimming skills and water safety interventions on drowning-related outcomes, caution is needed in making interferences based on drowning data.

Child drownings form a major public health issue in particularly LMICs (2, 3). Despite this observation, the above-synthesized evidence on drowning prevention programmes is characterized by a lack of data originating from these settings. Interventions proven effective in high income settings may not necessarily be transferrable to LMICs, either financially, logistically culturally or for other reasons. Several factors that determine the feasibility of rolling out these basic swimming skills and water safety programmes in LMICs are underexplored in the current body of evidence. Also, long-term retention of acquired aquatic skills or knowledge following the participation in an educational programme, although particularly important in settings where refresher courses and continued practice may not be feasible, is rarely explored. Redirecting research efforts toward low- and middle income contexts may be necessary to sustain the ongoing decline in drowning-related mortality and extend this progress, mainly observed in high-income countries (1), to settings with limited resources.

## 5 Conclusion

The evidence summarized in this review shows that training children in basic swimming skills and/or water safety (either isolated or as part of a broad injury prevention programme) may be an effective strategy to prevent death from drowning and improve children’s knowledge, skills and behaviour on water safety. This review update provides evidence on best implementation practices for swimming skills training, that could aid in guiding resource allocation to qualitative, sustainable programmes. In particular, prioritizing familiarization with aquatic environments and aquatic motor awareness over formal skill development might effectively enhance the development of swimming skills. Additional high-certainty evidence stemming from low- and middle-income settings is needed to tailor aquatic training programmes to local contexts that are highly impacted by child drownings.

## Data Availability

All relevant data presented in the systematic review are included in the article/Supplementary material, further inquiries can be directed to the corresponding author.

## Supplementary material

Appendix 1 PRISMA checklist.

Appendix 2 Database searches.

Appendix 3 Studies awaiting classification.

Appendix 4 Synthesis of findings tables.
