# Supplementary material for: Basic swimming or water safety skills training for drowning prevention in children: an updated systematic review
Source: Front Public Health. 2025 Dec 12;13:1698353. doi: 10.3389/fpubh.2025.1698353 (PMC12741065; doi:10.3389/fpubh.2025.1698353)
Supplement: Appendix 2 — Database searches. [file Table_2.docx]

**Appendix 2** Search strings for databases, clinical trial registers and grey literature sources

**Databases**

PubMed

1. "Education"[Mesh] OR "education"[Subheading] OR educat*[TIAB] OR teach*[TIAB] OR curricul*[TIAB] OR train*[TIAB] OR instruct*[TIAB] OR learn*[TIAB] OR course*[TIAB] OR lesson*[TIAB] OR program*[TIAB]

2. "Drowning"[Mesh] OR drown*[TIAB] OR “water submersion”[TIAB] OR “water submersions”[TIAB] OR “water immersion”[TIAB] OR “water immersions”[TIAB] OR "Swimming"[Mesh] OR swim*[TIAB] OR “water trauma”[TIAB] OR “water phobia”[TIAB] OR “water-related injury”[TIAB] OR aquaticity[TIAB] OR “water safety”[TIAB] OR aquatic[TIAB] OR “water skills”[TIAB] OR “water confidence”[TIAB] OR “water behavior”[TIAB] OR “water behaviour”[TIAB] OR “water-related behavior”[TIAB:~0] OR “water-related behaviour”[TIAB:~0] OR “water survival”[TIAB] OR “water exposure”[TIAB] OR “exposure to water”[TIAB] OR “water recovery”[TIAB] OR float*[TIAB] OR flotation*[TIAB] OR buoyan*[TIAB] OR “treading water”[TIAB] OR “tread water”[TIAB] OR “water treading”[TIAB] OR rescue skill*[TIAB] OR survival skill*[TIAB] OR safety skill*[TIAB] OR “side stroke”[TIAB:~0] OR backstroke[TIAB] OR “survival stroke”[TIAB:~0] OR “survival strokes”[TIAB:~0]

3. “Infant”[Mesh] OR “Child”[Mesh] OR “Adolescent”[Mesh] OR infant*[TIAB] OR toddler*[TIAB] OR child*[TIAB] OR kid[TIAB] OR kids[TIAB] OR youth[TIAB] OR youths[TIAB] OR juvenile*[TIAB] OR teenage*[TIAB] OR teen[TIAB] OR teens[TIAB] OR adolescent*[TIAB] OR “Students”[Mesh] OR student*[TIAB] OR pupil*[TIAB] OR “Schools”[Mesh] OR school*[TIAB] OR preschool*[TIAB]

4. 1-3 AND

Embase

1. 'education'/exp OR educat*:ab,ti OR teach*:ab,ti OR curricul*:ab,ti OR 'training'/exp OR train*:ab,ti OR instruct*:ab,ti OR 'learning'/exp OR learn*:ab,ti OR course*:ab,ti OR lesson*:ab,ti OR program*:ab,ti

2. 'drowning'/exp OR drown*:ab,ti OR ‘water submersion’:ab,ti OR ‘water submersions’:ab,ti OR ‘water immersion’:ab,ti OR ‘water immersions’:ab,ti OR 'swimming'/exp OR swim*:ab,ti OR ‘water trauma’:ab,ti OR ‘water phobia’:ab,ti OR ‘water-related injury’:ab,ti OR aquaticity:ab,ti OR ‘water safety’:ab,ti OR aquatic:ab,ti OR ‘water skills’:ab,ti OR ‘water confidence’:ab,ti OR ‘water behavior’:ab,ti OR ‘water behaviour’:ab,ti OR ‘water-related behavior’:ab,ti OR ‘water-related behaviour’:ab,ti OR ‘water survival’:ab,ti OR ‘water exposure’:ab,ti OR ‘exposure to water’:ab,ti OR ‘water recovery’:ab,ti OR float*:ab,ti OR flotation*:ab,ti OR buoyan*:ab,ti OR ‘treading water’:ab,ti OR ‘tread water’:ab,ti OR ‘water treading’:ab,ti OR (rescue NEXT/1 skill*):ab,ti OR (survival NEXT/1 skill*):ab,ti OR (safety NEXT/1 skill*):ab,ti OR ‘side stroke’:ab,ti OR backstroke:ab,ti OR ‘survival stroke’:ab,ti OR ‘survival strokes’:ab,ti

3. ‘juvenile’/exp OR infant*:ab,ti OR toddler*:ab,ti OR child*:ab,ti OR kid:ab,ti OR kids:ab,ti OR youth:ab,ti OR youths:ab,ti OR juvenile*:ab,ti OR teenage*:ab,ti OR teen:ab,ti OR teens:ab,ti OR adolescent*:ab,ti OR ‘student’/exp OR student*:ab,ti OR pupil*:ab,ti OR 'school'/exp OR school*:ab,ti OR preschool*:ab,ti

4. 1-3 AND

The Cochrane Library

1. [mh "Education"] OR educat*:ti,ab,kw OR teach*:ti,ab,kw OR curricul*:ti,ab,kw OR train*:ti,ab,kw OR instruct*:ti,ab,kw OR learn*:ti,ab,kw OR course*:ti,ab,kw OR lesson*:ti,ab,kw OR program*:ti,ab,kw

2. [mh “Drowning"] OR drown*:ti,ab,kw OR “water submersion”:ti,ab,kw OR “water submersions”:ti,ab,kw OR “water immersion”:ti,ab,kw OR “water immersions”:ti,ab,kw OR [mh “Swimming"] OR swim*:ti,ab,kw OR “water trauma”:ti,ab,kw OR “water phobia”:ti,ab,kw OR “water related injury”:ti,ab,kw OR aquaticity:ti,ab,kw OR “water safety”:ti,ab,kw OR aquatic:ti,ab,kw OR “water skills”:ti,ab,kw OR “water confidence”:ti,ab,kw OR “water behavior”:ti,ab,kw OR “water behaviour”:ti,ab,kw OR “water related behavior”:ti,ab,kw OR “water related behaviour”:ti,ab,kw OR “water survival”:ti,ab,kw OR “water exposure”:ti,ab,kw OR “exposure to water”:ti,ab,kw OR “water recovery”:ti,ab,kw OR float*:ti,ab,kw OR floatation*:ti,ab,kw OR buoyan*:ti,ab,kw OR “treading water”:ti,ab,kw OR “tread water”:ti,ab,kw OR “water treading”:ti,ab,kw OR (rescue NEXT skill*):ti,ab,kw OR (survival NEXT skill*):ti,ab,kw OR (safety NEXT skill*):ti,ab,kw OR “side stroke”:ti,ab,kw OR backstroke:ti,ab,kw OR “survival stroke”:ti,ab,kw OR “survival strokes”:ti,ab,kw

3. [mh “Infant”] OR [mh “Child”] OR [mh “Adolescent”] OR infant*:ti,ab,kw OR toddler*:ti,ab,kw OR child*:ti,ab,kw OR kid:ti,ab,kw OR kids:ti,ab,kw OR youth:ti,ab,kw OR youths:ti,ab,kw OR juvenile*:ti,ab,kw OR teenage*:ti,ab,kw OR teen:ti,ab,kw OR teens:ti,ab,kw OR adolescent*:ti,ab,kw OR [mh “Students”] OR student*:ti,ab,kw OR pupil*:ti,ab,kw OR [mh “Schools”] OR school*:ti,ab,kw OR preschool*:ti,ab,kw

4. 1-3 AND

Web of Science

1. TS=(“educat*” OR “teach*” OR “curricul*” OR “train*” OR “instruct*” OR “learn*” OR “course*” OR “lesson*” OR “program*”)

2. TS=(“drown*” OR “water submersion” OR “water submersions” OR “water immersion” OR “water immersions” OR “swim*” OR “water trauma” OR “water phobia” OR “water-related injury” OR “aquaticity” OR “water safety” OR “aquatic” OR “water skills” OR “water confidence” OR “water behavior” OR “water behaviour” OR “water-related behavior” OR “water-related behaviour” OR “water survival” OR “water exposure” OR “exposure to water” OR “water recovery” OR “float*” OR "flotation*" OR “buoyan*” OR “treading water” OR “tread water” OR “water treading” OR “rescue skill*” OR “survival skill*” OR “safety skill*” OR “side stroke” OR “backstroke” OR “survival stroke” OR “survival strokes”)

3. TS=(“infant*” OR “toddler*” OR “child*” OR “kid” OR “kids” OR “youth” OR “youths” OR “juvenile*” OR “teenage*” OR “teen” OR “teens” OR “adolescent*” OR “student*” OR “pupil*” OR “school*” OR “preschool*”)

4. 1-3 AND

ERIC (via OvidSP interface)

1. exp Education/ OR educat*.ti,ab. OR teach*.ti,ab. OR curricul*.ti,ab. OR train*.ti,ab. OR instruct*.ti,ab. OR learn*.ti,ab. OR course*.ti,ab. OR lesson*.ti,ab. OR program*.ti,ab.

2. drown*.ti,ab. OR water submersion.ti,ab. OR water submersions.ti,ab. OR water immersion.ti,ab. OR water immersions.ti,ab. OR swim*.ti,ab. OR water trauma.ti,ab. OR water phobia.ti,ab. OR water-related injury.ti,ab. OR aquaticity.ti,ab. OR water safety.ti,ab. OR aquatic.ti,ab. OR water skills.ti,ab. OR water confidence.ti,ab. OR water behavior.ti,ab. OR water behaviour.ti,ab. OR water-related behavior.ti,ab. OR water-related behaviour.ti,ab. OR water survival.ti,ab. OR water exposure.ti,ab. OR exposure to water.ti,ab. OR water recovery.ti,ab. OR float*.ti,ab. OR flotation*.ti,ab. OR buoyan*.ti,ab. OR treading water.ti,ab. OR tread water.ti,ab. OR water treading.ti,ab. OR rescue skill*.ti,ab. OR survival skill*.ti,ab. OR safety skill*.ti,ab. OR side stroke.ti,ab. OR backstroke.ti,ab. OR survival stroke.ti,ab. OR survival strokes.ti,ab.

3. exp Young Children/ OR Children/ OR exp Preadolescents/ OR exp Adolescents/ OR exp Youth/ OR infant*.ti,ab. OR toddler*.ti,ab. OR child*.ti,ab. OR kid.ti,ab. OR kids.ti,ab. OR youth.ti,ab. OR youths.ti,ab. OR juvenile*.ti,ab. OR teenage*.ti,ab. OR teen.ti,ab. OR teens.ti,ab. OR adolescent*.ti,ab. OR Students/ OR exp Secondary School Students/ OR exp High School Students/ OR exp Junior High School Students/ OR exp Middle School Students/ OR exp Elementary School Students/ OR student*.ti,ab. OR pupil*.ti,ab. OR exp Schools OR school*.ti,ab. OR preschool*.ti,ab.

4. 1-3 AND

**Clinical trials registers**

clinicaltrails.gov

Drowning OR (Swimming AND (Education OR educate OR educating OR course OR courses OR instruct OR instruction OR instructions OR instructing)

WHO International Clinical Trials Registry Platform

drown* OR (swim* AND (educat* OR course* OR instruct*))

**Grey literature sources**

The following grey literature sources were searched using search terms "drown", "drowning", "swim", "swimming", "water safety", "water training" and "water skills":

- Grey Literature Report (https://catalog.nyam.org/)
- OpenGrey (https://opengrey.eu/)
- 3ei Database of Impactful Evaluations (https://developmentevidence.3ieimpact.org/)
- WHO Library Database (https://kohahq.searo.who.int/)
- World Bank Documents and Reports (http://documents.worldbank.org/curated/en/home)
- EPPI Centre Databases (https://eppi.ioe.ac.uk/cms/Default.aspx?tabid=185)
  - Database of promoting Health Effectiveness Reviews (DoPHER)
  - Trials Register of Promoting Health Interventions (TRoPHI)
  - Bibliomap
  - Database of Education research
- Global Index Medicus (https://pesquisa.bvsalud.org/gim/advanced/?lang=en)
- Save the Children Resource Centre (https://resourcecentre.savethechildren.net/)
- Oxfam Open Repository (https://oxfamilibrary.openrepository.com/)
- UNICEF Research and Reports (https://www.unicef.org/eca/search)
