# Supplementary material for: Basic swimming or water safety skills training for drowning prevention in children: an updated systematic review
Source: Front Public Health. 2025 Dec 12;13:1698353. doi: 10.3389/fpubh.2025.1698353 (PMC12741065; doi:10.3389/fpubh.2025.1698353)
Supplement: Appendix 3 — Studies awaiting classification. [file Table_3.docx]

**Appendix 3** Studies awaiting classification

| **ID** | **Title** | **Reason** |
| --- | --- | --- |
| Jaafar, 2021 | The impact of the use of teaching technology in learning technical performance and accuracy of swimming skills | Population not specified. Authors were contacted twice but no response was received. |
| Rao, 2025 | A quality improvement project to increase provider-directed drowning prevention anticipatory guidance in the primary care clinic setting | Conference abstract without usable data. No published article identified. |
