# Supplementary material for: Basic swimming or water safety skills training for drowning prevention in children: an updated systematic review
Source: Front Public Health. 2025 Dec 12;13:1698353. doi: 10.3389/fpubh.2025.1698353 (PMC12741065; doi:10.3389/fpubh.2025.1698353)
Supplement: Appendix 4 — Synthesis of findings tables. [file Table_4.docx]

**Appendix 4** Synthesis of findings and GRADE tables. Adapted from the WHO Guideline on the prevention of drowning through provision of day-care, and basic swimming and water safety skills, 2021 (pp 72-99). WHO is not responsible for the content or accuracy of this adaptation. <https://www.who.int/publications/i/item/9789240030008>, accessed on 01/04/2025)

[Part 1 – Basic swimming skills training (in water) 3](#_Toc204760406)

[Comparison 1.1. An educational programme for basic swimming skills with in-water training vs no educational programme for basic swimming skills 3](#_Toc204760407)

[Comparison 1.2. An educational programme for basic swimming skills using motility stories vs a traditional educational programme for basic swimming skills 5](#_Toc204760408)

[Comparison 1.3. An educational programme for basic swimming skills vs an educational programme for formal swimming techniques 6](#_Toc204760409)

[Comparison 1.4. An educational programme for basic swimming skills using aquatic motor competence training vs a traditional educational programme for basic swimming skills 9](#_Toc204760410)

[Comparison 1.5. Tec Pa educational programme for basic swimming skills vs a traditional educational programme for basic swimming skills 10](#_Toc204760411)

[Comparison 1.6. An educational programme for basic swimming skills using aquatic motor games vs a traditional educational programme for basic swimming skills 12](#_Toc204760412)

[Comparison 1.7. An educational programme for basic swimming skills with daily training vs an educational programme for basic swimming skills with weekly training 13](#_Toc204760413)

[Comparison 1.8. An educational programme for basic swimming skills using buoyancy aids vs an educational programme for basic swimming skills without the use of buoyancy aids 14](#_Toc204760414)

[Comparison 1.9. An educational programme for basic swimming skills using goggles and snorkels vs an educational programme for basic swimming skills without the use of goggles and snorkels 18](#_Toc204760415)

[Comparison 1.10. An educational programme for basic swimming skills using video-taped feedback vs an educational programme for basic swimming skills using auditory feedback 22](#_Toc204760416)

[Comparison 1.11. An educational programme for basic swimming skills in shallow water vs an educational programme for basic swimming skills in deep water 23](#_Toc204760417)

[Part 2 - Water safety training (out-of-water) 27](#_Toc204760418)

[Comparison 2.1. An educational programme for water safety skills without in-water training vs no educational programme for water safety skills 27](#_Toc204760419)

[Comparison 2.2. An educational programme for water safety skills without in-water training using virtual reality vs an educational programme for water safety skills using another mode of delivery 31](#_Toc204760420)

[Part 3 - Water safety training (out-of-water) as part of a broad educational programme on injury prevention 32](#_Toc204760421)

[Comparison 3.1. A broad educational programme containing out-of-water water safety skills training vs no broad educational programme for water safety skills training 32](#_Toc204760422)

[Comparison 3.2. A multimodal educational programme for water safety skills training (out-of-water) vs handbook education only 35](#_Toc204760423)

[Part 4 – Combined basic swimming skills training (in water) and water safety training (out-of-water) 36](#_Toc204760424)

[Comparison 4.1. An educational programme combining basic swimming skills training and water safety skills with out-of-water training vs no educational programme for water safety skills 36](#_Toc204760425)

[Comparison 4.2. A 12-week educational programme combining basic swimming skills training and water safety skills with out-of-water training vs an 8-week educational programme combining basic swimming skills training and water safety skills with out-of-water training 42](#_Toc204760426)

# Part 1 – Basic swimming skills training (in water)

## Comparison 1.1. An educational programme for basic swimming skills with in-water training vs no educational programme for basic swimming skills

| N° of studies | Study design | Risk of bias | Inconsistency | Indirectness | Imprecision | Other considerations | Number of participants | | Effect | | Certainty |
| --- | --- | --- | --- | --- | --- | --- | --- | --- | --- | --- | --- |
|  |  |  |  |  |  |  | Intervention | Control | Relative (95%CI) | Absolute (95%CI) |  |
| Drowning-related mortality: children aged 0-4 years | | | | | | | | | | | |
| 2^1,2^ | Observational: case-control studies | serious^a^ | not serious | not serious | serious^b^ | none | 61 cases, 134 controls | | aOR: 0.12  (0.01 to 0.97) | - | 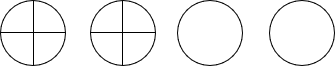 Low |
|  |  |  |  |  |  |  | 64 cases, 128 controls | | aOR: 1.8 (1.1 to 5.5) | - |  |
|  |  |  |  |  |  |  | aOR were not combined in a meta-analysis since the direction of the reported results differed between studies. However, both showed results in favour of swimming skills training. | | | |  |
| Drowning-related mortality: children aged 5-14 years | | | | | | | | | | | |
| 1^2^ | Observational: case-control study | serious^a^ | not serious | not serious | very serious^c^ | none | 69 cases, 138 controls | | -- (-- to --)  (p > 0.05)^d^ | - | 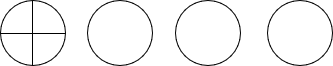 Very low |
| Water safety skills: Swimming performance in domain "Locomotion: front" after 8 months of intervention | | | | | | | | | | | |
| 1^3^ | Observational: cohort study | very serious^e^ | not serious | not serious | serious^b^ | none | 30 | 64 | - | MD: 3.90 higher (-- to --)^f^  (p < 0.05) | 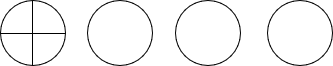 Very low |
| Water safety skills: Swimming performance in domain "Locomotion: back" after 8 months of intervention | | | | | | | | | | | |
| 1^3^ | Observational: cohort study | very serious^e^ | not serious | not serious | serious^b^ | none | 30 | 64 | - | MD: 3.40 higher (-- to --)^f^ (p < 0.05) | 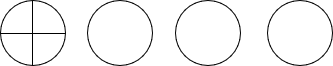 Very low |
| Water safety skills: Swimming skills in domain "Kicking" after 8 months of intervention | | | | | | | | | | | |
| 1^3^ | Observational: cohort study | very serious^e^ | not serious | not serious | serious^b^ | none | 30 | 64 | - | MD: 3.60 higher (-- to --)^f^ (p < 0.05) | 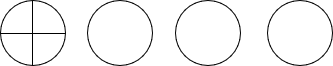 Very low |
| Water safety skills: Swimming performance in domain "Entry: jump" after 8 months of intervention | | | | | | | | | | | |
| 1^3^ | Observational: cohort study | very serious^e^ | not serious | not serious | serious^b^ | none | 30 | 64 | - | MD: 3.60 higher (-- to --)^f^ (p < 0.05) | 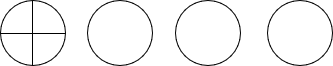 Very low |
| Water safety skills: Swimming performance in domain "Diving" after 8 months of intervention | | | | | | | | | | | |
| 1^3^ | Observational: cohort study | very serious^e^ | not serious | not serious | serious^b^ | none | 30 | 64 | - | MD: 0.40 higher (-- to --)^f^ (p < 0.05) | 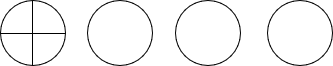 Very low |
| Water safety skills: Swimming performance in domain "Ring pick-up" after 8 months of intervention | | | | | | | | | | | |
| 1^3^ | Experimental: non-RCT | very serious^e^ | not serious | not serious | serious^b^ | none | 30 | 64 | - | MD: 1.10 higher (-- to --)^f^ (p < 0.05) | 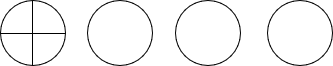 Very low |
| Water safety skills: Self-reported ability to swim 200 metres, 2 years after the intervention | | | | | | | | | | | |
| 1^4^ | Observational: cohort study | very serious^g^ | not serious | not serious | serious^h^ | none | --/1695 (77.0%) | --/1773 (78.2%) | aOR: 1.03  (0.84 to 1.27) | - | 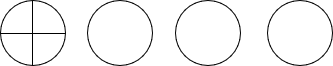 Very low |

**References**

1. Yang, L., Nong, Q.Q., Li, C.L., Feng, Q.M., & Lo, S.K. (2007). Risk factors for childhood drowning in rural regions of a developing country: a case-control study. Injury Prevention, 13(3), 178–182.
2. Brenner, R.A., Taneja, G.S., Haynie, D.L., Trumble, A.C., Qian, C., Klinger, R.M., & Klebanoff, M.A. (2009). Association between swimming lessons and drowning in childhood: A case-control study. Archives of Pediatrics & Adolescent Medicine, 163(3): 203–210.
3. Erbaugh, S.J. (1986). Effects of aquatic training on swimming skill development of preschool children. Perceptual and Motor Skills, 62(2): 439–446.
4. Pilgaard, F.I.H., Östergren, P-O., Olin, A., Kling, S., Albin, M., & Björk, J. (2020). Socioeconomic differences in swimming ability among children in Malmö, southern Sweden: Initial results from a community-level intervention. Scandinavian Journal of Public Health, 48(5), 495-501.

**Notes**

a. Moderate risk of bias due to confounding, and moderate risk of bias in classification of participants into study (potential recall bias) (ROBINS-I).
b. Limited sample size.
c. Downgraded by 2 levels for imprecision due to a limited sample size and a lack of data prohibiting us to assess whether the 95%CI contained both appreciable harm and benefit (set at aOR > 1.25 and aOR < 0.75, respectively)
d. Effect measure and 95% confidence interval was not reported in the study and could not be calculated due to missing raw data.
e. Serious risk of bias due to confounding, and moderate risk of bias in measurement of outcomes (ROBINS-I).
f. 95% confidence interval was not reported in the study and could not be calculated due to multiple comparisons.
g. Serious risk of bias due to confounding (ROBINS-I).
h. Large variability of results.

## Comparison 1.2. An educational programme for basic swimming skills using motility stories vs a traditional educational programme for basic swimming skills

| N° of studies | Study design | Risk of bias | Inconsistency | Indirectness | Imprecision | Other considerations | | Number of participants | | | | Effect | | | Certainty |
| --- | --- | --- | --- | --- | --- | --- | --- | --- | --- | --- | --- | --- | --- | --- | --- |
|  |  |  |  |  |  |  |  | Intervention | | Control | | Relative (95%CI) | | Absolute (95%CI) |  |
| Water safety skills: aquatic motor competence, immediately after intervention | | | | | | | | | | | | | | | |
| 1^1^ | Experimental: non-RCT | very serious^a^ | not serious | not serious | serious^b^ | none | 8 | | 8 | | - | | MD: 0.07 higher (0.26 lower to 0.40 higher) | | 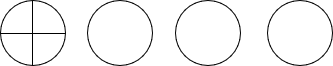 Very low |

**References**

1. Moreno-Murcia, J., Hernandez, E.H., Polo, R., Lopez, E., Carbonell, B., & Meseguer, S. (2016). The effect of stories on real and perceived aquatic competence in preschoolers. Revista Internacional de Medicina y Ciencias de la Actividad Fisica y del Deporte, 16(61), 127-138.

**Notes**

a. Serious risk of bias due to confounding, and moderate risk of bias in measurement of outcome (ROBINS-I).
c. Limited sample size.

## Comparison 1.3. An educational programme for basic swimming skills vs an educational programme for formal swimming techniques

| N° of studies | Study design | Risk of bias | Inconsistency | Indirectness | Imprecision | Other considerations | Number of participants | | Effect | | Certainty |
| --- | --- | --- | --- | --- | --- | --- | --- | --- | --- | --- | --- |
|  |  |  |  |  |  |  | Intervention | Control | Relative (95%CI) | Absolute (95%CI) |  |
| Water safety skills: aquatic skills in domain 'Water entry" (Scale from 1 to 3), immediately after the intervention | | | | | | | | | | | |
| 1^1^ | Experimental: RCT | very serious^a^ | not serious | not serious | serious^b^ | none | 17 | 14 | - | MD: 0.00 (not estimable) | 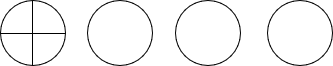 Very low |
| Water safety skills: aquatic skills in domain 'Water orientation and adjustment at ventral position" (Scale from 1 to 3), immediately after the intervention | | | | | | | | | | | |
| 1^1^ | Experimental: RCT | very serious^a^ | not serious | not serious | serious^b^ | none | 17 | 14 | - | MD: 0.33 higher (0.01 lower to 0.67 higher) | 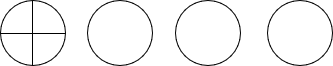 Very low |
| Water safety skills: aquatic skills in domain "Breath control – immersion of the face and eye opening" (Scale from 1 to 5), immediately after the intervention | | | | | | | | | | | |
| 1^1^ | Experimental: RCT | very serious^a^ | not serious | not serious | serious^b^ | none | 17 | 14 | - | MD: 0.11 higher (0.61 lower to 0.83 higher) | 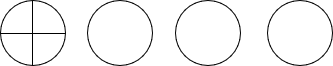 Very low |
| Water safety skills: aquatic skills in domain "Horizontal buoyancy" (Scale from 1 to 4), immediately after the intervention | | | | | | | | | | | |
| 1^1^ | Experimental: RCT | very serious^a^ | not serious | not serious | very serious^c^ | none | 17 | 14 | - | MD: 0.68 higher (0.01 to 1.35 higher) | 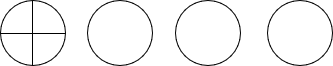 Very low |
| Water safety skills: aquatic skills in domain "Body position at ventral gliding" (Scale from 1 to 4), immediately after the intervention | | | | | | | | | | | |
| 1^1^ | Experimental: RCT | very serious^a^ | not serious | not serious | serious^b^ | none | 17 | 14 | - | MD: 0.25 higher (0.60 lower to 1.10 higher) | 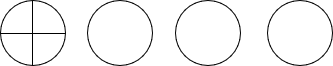 Very low |
| Water safety skills: aquatic skills in domain "Body position at dorsal gliding" (Scale from 1 to 4), immediately after the intervention | | | | | | | | | | | |
| 1^1^ | Experimental: RCT | very serious^a^ | not serious | not serious | serious^b^ | none | 17 | 14 | - | MD: 0.68 higher (0.08 to 1.28 higher) | 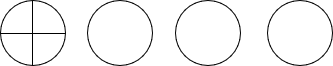 Very low |
| Water safety skills: aquatic skills in domain "Body position at longitudinal rotation in gliding" (Scale from 1 to 3), immediately after the intervention | | | | | | | | | | | |
| 1^1^ | Experimental: RCT | very serious^a^ | not serious | not serious | serious^b^ | none | 17 | 14 | - | MD: 0.28 higher (0.12 lower to 0.68 higher) | 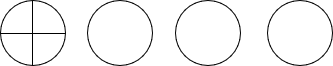 Very low |
| Water safety skills: aquatic skills in domain "Body position at front and back somersaults" (Scale from 1 to 4), immediately after the intervention | | | | | | | | | | | |
| 1^1^ | Experimental: RCT | very serious^a^ | not serious | not serious | serious^b^ | none | 17 | 14 | - | MD: 0.19 higher (0.51 lower to 0.89 higher) | 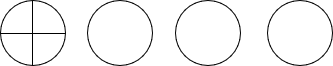 Very low |
| Water safety skills: aquatic skills in domain "Leg kick with breath control at ventral body position with flutter boards" (Scale from 1 to 4), immediately after the intervention | | | | | | | | | | | |
| 1^1^ | Experimental: RCT | very serious^a^ | not serious | not serious | serious^b^ | none | 17 | 14 | - | MD: 0.27 higher (0.44 lower to 0.98 higher) | 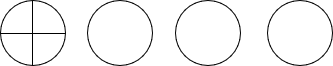 Very low |
| Water safety skills: aquatic skills in domain "Leg kick with breath control at ventral body position without flutter device" (Scale from 1 to 4), immediately after the intervention | | | | | | | | | | | |
| 1^1^ | Experimental: RCT | very serious^a^ | not serious | not serious | serious^b^ | none | 17 | 14 | - | MD: 0.76 higher (0.10 to 1.42 higher) | 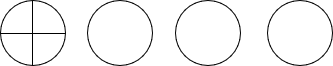 Very low |
| Water safety skills: aquatic skills in domain "Leg kick with breath control at dorsal body position with flutter boards" (Scale from 1 to 4), immediately after the intervention | | | | | | | | | | | |
| 1^1^ | Experimental: RCT | very serious^a^ | not serious | not serious | serious^b^ | none | 17 | 14 | - | MD: 0.71 higher (0.03 to 1.39 higher) | 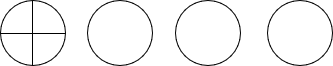 Very low |
| Water safety skills: aquatic skills in domain "Leg kick with breath control at dorsal body position without flutter boards" (Scale from 1 to 4), immediately after the intervention | | | | | | | | | | | |
| 1^1^ | Experimental: RCT | very serious^a^ | not serious | not serious | serious^b^ | none | 17 | 14 | - | MD: 0.69 higher (0.12 to 1.26 higher) | 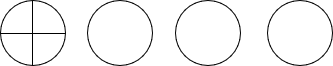 Very low |
| Water safety skills: aquatic skills in domain "Feet-first entry" (Scale from 1 to 3), immediately after the intervention | | | | | | | | | | | |
| 1^1^ | Experimental: RCT | very serious^a^ | not serious | not serious | serious^b^ | none | 17 | 14 | - | MD: 0.51 higher (0.21 to 0.81 higher) | 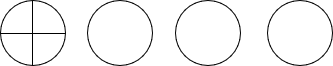 Very low |
| Water safety skills: aquatic skills in domain "Head-first entry" (Scale from 1 to 3), immediately after the intervention | | | | | | | | | | | |
| 1^1^ | Experimental: RCT | very serious^a^ | not serious | not serious | serious^b^ | none | 17 | 14 | - | MD: 0.06 higher (0.42 lower to 0.54 higher) | 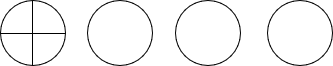 Very low |
| Water safety skills: aquatic skills in domain "Autonomous in deep pool (legs and arms displacement)" (Scale from 1 to 3), immediately after the intervention | | | | | | | | | | | |
| 1^1^ | Experimental: RCT | very serious^a^ | not serious | not serious | serious^b^ | none | 17 | 14 | - | MD: 0.21 higher (0.23 lower to 0.65 higher) | 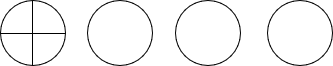 Very low |
| Water safety skills: aquatic skills in domain "Vertical buoyancy" (Scale from 1 to 5), immediately after the intervention | | | | | | | | | | | |
| 1^1^ | Experimental: RCT | very serious^a^ | not serious | not serious | serious^b^ | none | 17 | 14 | - | MD: 0.47 higher (0.13 lower to 1.07 higher) | 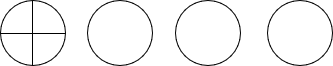 Very low |
| Water safety skills: aquatic skills in domain "Deep-water immersion" (Scale from 1 to 4), immediately after the intervention | | | | | | | | | | | |
| 1^1^ | Experimental: RCT | very serious^a^ | not serious | not serious | serious^b^ | none | 17 | 14 | - | MD: 1.31 higher (0.42 to 2.20 higher) | 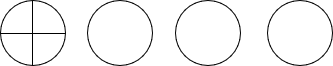 Very low |

**References**

1. Moura, O.M., Marinho, D.A., Forte, P., Fail, L.B., & Neiva, H.P. (2021). School-based swimming lessons enhance specific skills and motor coordination in children: the comparison between two interventions. Motricidade, 17(4): 367-374.

**Notes**

a. High risk of bias in the measurement of the outcome, some concerns on the randomization process, and some concerns on the selection of the reported results (Cochrane Risk of Bias-2).
b. Limited sample size.
c. Downgraded by 2 levels for imprecision due to a limited sample size and a large variability of results.

## Comparison 1.4. An educational programme for basic swimming skills using aquatic motor competence training vs a traditional educational programme for basic swimming skills

| N° of studies | Study design | | Risk of bias | Inconsistency | Indirectness | Imprecision | Other considerations | Number of participants | | Effect | | Certainty |
| --- | --- | --- | --- | --- | --- | --- | --- | --- | --- | --- | --- | --- |
|  |  |  |  |  |  |  |  | Intervention | Control | Relative (95%CI) | Absolute (95%CI) |  |
| Water safety skills: aquatic strokes score (/66), immediately after the intervention | | | | | | | | | | | | |
| 1^1^ | Experimental: (cluster-)RCT | very serious^a^ | | not serious | not serious | serious^b^ | none | 54 | 53 | - | MD: 11.7 higher (4.03 to 19.35 higher) | 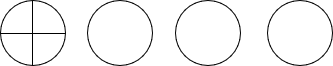 Very low |
| Water safety skills: aquatic skills score (/72), immediately after the intervention | | | | | | | | | | | | |
| 1^1^ | Experimental: (cluster-)RCT | very serious^a^ | | not serious | not serious | serious^b^ | none | 54 | 53 | - | MD: 0.00 (4.12 lower to 4.12 higher) | 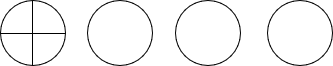 Very low |
| Water safety skills: aquatic motor performance score (/138), immediately after the intervention | | | | | | | | | | | | |
| 1^1^ | Experimental: (cluster-)RCT | very serious^a^ | | not serious | not serious | serious^b^ | none | 54 | 53 | - | MD: 13.5 higher (0.23 lower to 27.23 higher) | 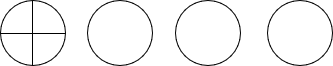 Very low |

**References**

1. Pratt, N.A., Duncan, M.J., & Oxford, S.W. (2023). The effects of a 6-week swimming intervention on gross motor development in primary school children. Children, 11, 1.

**Notes**

a. High risk of bias in measurement of the outcome, some concerns on the randomization process, and some concerns on the selection of the reported results (Cochrane Risk of Bias-2).
b. Limited sample size.

## Comparison 1.5. Tec Pa educational programme for basic swimming skills vs a traditional educational programme for basic swimming skills

| N° of studies | Study design | | Risk of bias | Inconsistency | Indirectness | Imprecision | | Other considerations | Number of participants | | Effect | | | | Certainty |
| --- | --- | --- | --- | --- | --- | --- | --- | --- | --- | --- | --- | --- | --- | --- | --- |
|  |  |  |  |  |  |  |  |  | Intervention | Control | Relative (95%CI) | | Absolute (95%CI) | |  |
| Water safety skills: number of children successfully performing "start", at the end of the intervention programme | | | | | | | | | | | | | | | |
| 1^1^ | | Experimental: RCT | very serious^a^ | not serious | not serious | serious^b^ | none | | 12/12 (100.0%) | 7/11 (63.6%) | | RR: 1.54 (-- to --) (p < 0.05)^c^ | - | 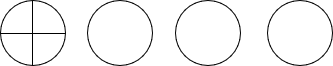 Very low | |
| Water safety skills: number of children successfully performing "sink" on first attempt, at the end of the intervention programme | | | | | | | | | | | | | | | |
| 1^1^ | | Experimental: RCT | very serious^a^ | not serious | not serious | serious^b^ | none | | 9/12 (75.0%) | 10/11 (90.9%) | | RR: 0.83 (-- to --) (p > 0.05)^c^ | - | 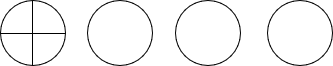 Very low | |
| Water safety skills: number of children successfully performing "sink" on second attempt, at the end of the intervention programme | | | | | | | | | | | | | | | |
| 1^1^ | | Experimental: RCT | very serious^a^ | not serious | not serious | serious^b^ | none | | 9/12 (75.0%) | 11/11 (100.0%) | | RR: 0.76 (-- to --) (p > 0.05)^c^ | - | 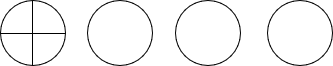 Very low | |
| Water safety skills: time needed to execute "start" and two "sink" attempts (s), at the end of the intervention programme | | | | | | | | | | | | | | | |
| 1^1^ | | Experimental: RCT | very serious^a^ | not serious | not serious | serious^b^ | none | | 12 | 11 | | - | MD: 3.6 lower  (-- to --)^d^ (p > 0.05) | 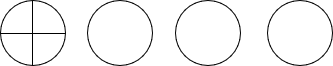 Very low | |
| Water safety skills: time needed to swim 35 m with a kickboard (s), at the end of the intervention programme | | | | | | | | | | | | | | | |
| 1^1^ | | Experimental: RCT | very serious^a^ | not serious | not serious | serious^b^ | none | | 12 | 11 | | - | MD: 4.5 lower (-- to --)^d^ (p > 0.05) | 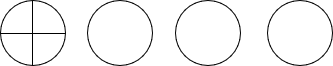 Very low | |
| Water safety skills: backstroke swimming technique, at the end of the intervention programme | | | | | | | | | | | | | | | |
| 1^1^ | | Experimental: RCT | very serious^a^ | not serious | not serious | serious^b^ | none | | 12 | 11 | | - | MD: 3.2 higher (1.93 to 4.48 higher) | 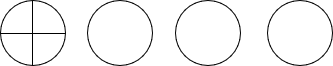 Very low | |
| Water safety skills: breaststroke swimming technique, at the end of the intervention programme | | | | | | | | | | | | | | | |
| 1^1^ | | Experimental: RCT | very serious^a^ | not serious | not serious | serious^b^ | none | | 12 | 11 | | - | MD: 2.6 higher (0.15 to 5.03 higher) | 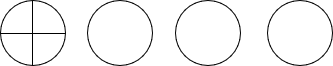 Very low | |

**References**

1. Papadimitriou, K., & Loupos, D. (2021). The Effect of an Alternative Swimming Learning Program on Skills, Technique, Performance, and Salivary Cortisol Concentration at Primary School Ages Novice Swimmers. Healthcare, 9, 1234.

**Notes**

a. High risk of bias in the randomization process, high risk of bias due to missing outcome data, and some concerns on the selection of the reported results (Cochrane Risk of Bias-2).
b. Low number of events.
c. 95% confidence interval was not reported in the study and could not be calculated due to multiple comparisons. Reported p-value results from a Fisher's exact analysis executed by the study authors.
d. 95% confidence interval was not reported in the study and could not be calculated due to multiple comparisons. Not possible to assess whether 95%CI contains both appreciable harm and benefits.

## Comparison 1.6. An educational programme for basic swimming skills using aquatic motor games vs a traditional educational programme for basic swimming skills

| N° of studies | Study design | Risk of bias | Inconsistency | Indirectness | Imprecision | Other considerations | Number of participants | | Effect | | Certainty |
| --- | --- | --- | --- | --- | --- | --- | --- | --- | --- | --- | --- |
|  |  |  |  |  |  |  | Intervention | Control | Relative (95%CI) | Absolute (95%CI) |  |
| Water safety skills: aquatic motor competence in domain "familiarization", Δ post-pre | | | | | | | | | | | |
| 1^1^ | Experimental: RCT | very serious^a^ | not serious | not serious | very serious^b^ | none | 8 | 9 | - | MD: 0.15 higher (-- to --)^c^ (p > 0.05) | 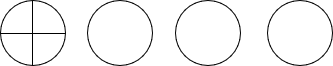 Very low |
| Water safety skills: aquatic motor competence in domain "immersion", Δ post-pre | | | | | | | | | | | |
| 1^1^ | Experimental: RCT | very serious^a^ | not serious | not serious | very serious^b^ | none | 8 | 9 | - | MD: 0.42 higher (-- to --)^c^ (p < 0.05) | 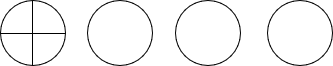 Very low |

**References**

1. Simon-Piqueras, J.A., Prieto-Ayuso, A., Gopez-Moreno, E., Martinez-Lopez, M., & Gil-Madrona, P. (2022). Evaluation of a program of aquatic motor games in the improvement of motor competence in children from 4 to 5 years old. Children, 9, 1141.

**Notes**

a. High risk of bias in the measurement of the outcome, some concerns on the randomization process, and some concerns on the selection of the reported results (Cochrane Risk of Bias-2).
b. Downgraded by 2 levels for imprecision due to a limited sample size and a lack of data prohibiting us to assess whether the 95%CI contained both appreciable harm and benefit.
c. 95% confidence interval was not reported in the study and could not be calculated due to a lack of data.

## Comparison 1.7. An educational programme for basic swimming skills with daily training vs an educational programme for basic swimming skills with weekly training

| N° of studies | Study design | Risk of bias | Inconsistency | Indirectness | Imprecision | Other considerations | Number of participants | | Effect | | | Certainty |
| --- | --- | --- | --- | --- | --- | --- | --- | --- | --- | --- | --- | --- |
|  |  |  |  |  |  |  | Intervention | Control | Relative (95%CI) | Absolute (95%CI) | |  |
| Water safety skills: front crawl swimming skills rate of improvement | | | | | | | | | | | | |
| 1^1^ | Experimental: non-RCT | very serious^a^ | not serious | not serious | very serious^b^ | none | 17 | 16 | - | -- (-- to --) (p > 0.05)^c^ | 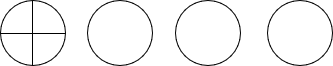 Very low | |

**References**

1. Bradley, S.M., Parker, H.E., & Blanksby, B.A. (1996). Learning front-crawl swimming by daily or weekly lesson schedules. Pediatric Exercise Science, 8(1), 27–36.

**Notes**

a. Serious risk of bias due to confounding, serious risk of bias in the measurement of the outcomes and moderate risk of bias due to missing data (ROBINS-I).
b. Downgraded by 2 levels for imprecision due to a limited sample size and a lack of data prohibiting us to assess whether the 95%CI contained both appreciable harm and benefit.
c. Effect measure and 95% confidence interval were not reported in the study and could not be calculated due to a lack of data.

## Comparison 1.8. An educational programme for basic swimming skills using buoyancy aids vs an educational programme for basic swimming skills without the use of buoyancy aids

| N° of studies | Study design | Risk of bias | Inconsistency | Indirectness | Imprecision | Other considerations | Number of participants | | Effect | | Certainty |
| --- | --- | --- | --- | --- | --- | --- | --- | --- | --- | --- | --- |
|  |  |  |  |  |  |  | Intervention | Control | Relative (95%CI) | Absolute (95%CI) |  |
| Water safety skills: aquatic readiness in domain "propulsion" (Scale from 1 to 10), at the end of the intervention | | | | | | | | | | | |
| 1^1^ | Experimental: RCT | very serious^a^ | not serious | not serious | serious^b^ | none | 11 | 13 | - | MD: 0.1 lower (0.78 lower to 0.58 higher) | 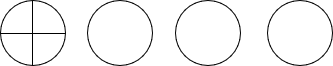 Very low |
| Water safety skills: aquatic readiness in domain "breathing and surface diving" (Scale from 1 to 10), at the end of the intervention | | | | | | | | | | | |
| 1^1^ | Experimental: RCT | very serious^a^ | not serious | not serious | serious^b^ | none | 11 | 13 | - | MD: 1.1 lower (2.45 lower to 0.25 higher) | 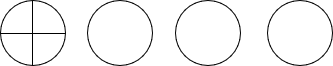 Very low |
| Water safety skills: aquatic readiness in domain "water entry" (Scale from 1 to 10), at the end of the intervention | | | | | | | | | | | |
| 1^1^ | Experimental: RCT | very serious^a^ | not serious | not serious | serious^b^ | none | 11 | 13 | - | MD: 0.7 lower (3.77 lower to 2.37 higher) | 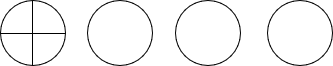 Very low |
| Water safety skills: movement distance in water during free play (m), at the end of the intervention | | | | | | | | | | | |
| 1^1^ | Experimental: RCT | very serious^c^ | not serious | not serious | serious^b^ | none | 11 | 13 | - | MD: 2.2 higher (2.62 lower to 7.02 higher) | 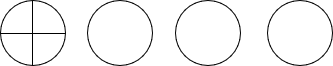 Very low |
| Water safety skills: movement distance on land during free play (m), at the end of the intervention | | | | | | | | | | | |
| 1^1^ | Experimental: RCT | very serious^c^ | not serious | not serious | serious^b^ | none | 11 | 13 | - | MD: 5.1 higher (3.55 lower to 13.75 higher) | 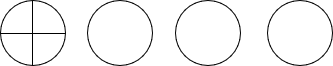 Very low |
| Water safety skills: floating performance in prone position, at the end of the intervention | | | | | | | | | | | |
| 1^2^ | Experimental: RCT | very serious^a^ | not serious | not serious | serious^b^ | none | 50 | 60 | - | MD: 0.3 lower  (1.01 lower to 0.41 higher) | 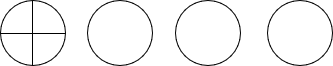 Very low |
| Water safety skills: floating performance in supine position, at the end of the intervention | | | | | | | | | | | |
| 1^2^ | Experimental: RCT | very serious^a^ | not serious | not serious | serious^b^ | none | 50 | 60 | - | MD: 0.5 lower  (1.34 lower to 0.34 higher) | 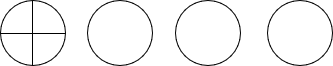 Very low |
| Water safety skills: gliding performance, at the end of the intervention | | | | | | | | | | | |
| 1^2^ | Experimental: RCT | very serious^a^ | not serious | not serious | serious^b^ | none | 50 | 60 | - | MD: 0.3 lower (0.69 lower to 0.09 higher) | 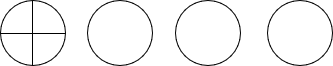 Very low |
| Water safety skills: leg kicking performance (Scale from 1 to 5), at the end of the intervention | | | | | | | | | | | |
| 1^3^ | Experimental: RCT | very serious^a^ | not serious | not serious | serious^b^ | none | 40 | 59 | - | MD: 0.1 lower (0.47 lower to 0.27 higher) | 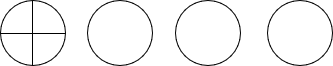 Very low |
| Water safety skills: arm propulsion performance (Scale from 1 to 5), at the end of the intervention | | | | | | | | | | | |
| 1^3^ | Experimental: RCT | very serious^a^ | not serious | not serious | serious^b^ | none | 40 | 59 | - | MD: 0.1 lower (0.30 lower to 0.1 higher) | 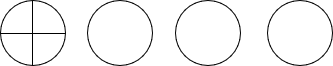 Very low |
| Water safety skills: arm recovery performance (Scale from 1 to 5), at the end of the intervention | | | | | | | | | | | |
| 1^3^ | Experimental: RCT | very serious^a^ | not serious | not serious | serious^b^ | none | 40 | 59 | - | MD: 0.1 lower  (0.38 lower to 0.18 higher) | 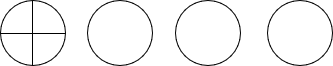 Very low |
| Water safety skills: overall swimming performance (Scale from 1 to 5), at the end of the intervention | | | | | | | | | | | |
| 1^3^ | Experimental: RCT | very serious^a^ | not serious | not serious | serious^b^ | none | 40 | 59 | - | MD: 0.1 lower (0.40 lower to 0.20 higher) | 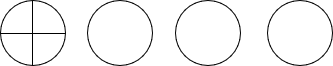 Very low |
| Water safety skills: front crawl performance in domain "leg action" (Scale from 1 to 14), at the end of the intervention | | | | | | | | | | | |
| 1^4^ | Experimental: RCT | serious^d^ | not serious | not serious | very serious^e^ | none | 10 | 8 | - | MD: 1.60 higher (-- to --)^f^ (p > 0.05) | 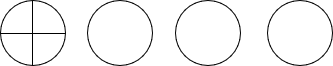 Very low |
| Water safety skills: front crawl performance in domain "leg action during arm stroking" (Scale from 1 to 14), at the end of the intervention | | | | | | | | | | | |
| 1^4^ | Experimental: RCT | serious^d^ | not serious | not serious | very serious^e^ | none | 10 | 8 | - | MD: 1.53 higher (-- to --)^f^ (p > 0.05) | 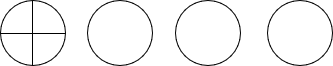 Very low |
| Water safety skills: front crawl performance in domain "arm action" (Scale from 1 to 14), at the end of the intervention | | | | | | | | | | | |
| 1^4^ | Experimental: RCT | serious^d^ | not serious | not serious | very serious^e^ | none | 10 | 8 | - | MD: 1.00 higher (-- to --)^f^ (p > 0.05) | 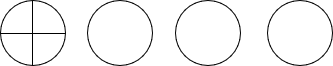 Very low |
| Water safety skills: aquatic capacity, at the end of the intervention | | | | | | | | | | | |
| 1^5^ | Experimental: RCT | very serious^g^ | not serious | not serious | very serious^e^ | none | This study compared the use of 3 buoyancy aids with each other: kiflot vs cuffs vs flotation belt (n = 6 for each). p=0.078 in the chi square analysis over the 3 intervention groups. | | | | 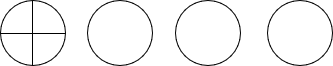 Very low |
| Water safety behaviour: number of jumps performed during free play, at the end of the intervention | | | | | | | | | | | |
| 1^1^ | Experimental: RCT | very serious^a^ | not serious | not serious | serious^b^ | none | 11 | 13 | - | MD: 1.5 lower (3.94 lower to 0.94 higher) | 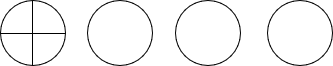 Very low |
| Water safety behaviour: number of surface dives performed during free play, at the end of the intervention | | | | | | | | | | | |
| 1^1^ | Experimental: RCT | very serious^a^ | not serious | not serious | serious^b^ | none | 11 | 13 | - | MD: 3.4 lower (5.43 to 1.37 lower) | 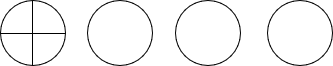 Very low |
| Water safety behaviour: children requesting a floatation aid during free play, at the end of the intervention | | | | | | | | | | | |
| 1^1^ | Experimental: RCT | very serious^a^ | not serious | not serious | serious^h^ | none | 4/11 (36.4%) | 0/13 (0.0%) | RR: 10.5 (0.63 to 175.83) | - | 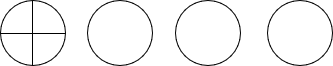 Very low |
| Water safety behaviour: voluntary entry with no fear of water, at the end of the intervention | | | | | | | | | | | |
| 1^4^ | Experimental: RCT | serious^d^ | not serious | not serious | very serious^j^ | none | 8/9 (88.9%) | 5/5 (100.0%) | RR: 0.93 (0.65 to 1.32) | - | 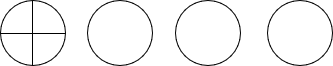 Very low |

**References**

1. Kjendlie, P-L., & Mendritzki, M. (2012). Movement patterns in free water play after swimming lessons with flotation aids. International Journal of Aquatic Research and Education, 6: 149-155.
2. Kjendlie, P-L. (2009a). No effect of using flotation suits in gliding and floating abilities of advanced beginners in swimming teaching. 14^th^ Annual ECSS Congress Oslo, June 24-27, 2009.
3. Kjendlie, P-L. (2009b). Swimming abilities are not enhanced by using a floatation suit for advanced beginners in deep water swimming teaching. 15^th^ Annual Congress ECSS, Oslo June 23-27, 2009.
4. Parker, H.E., Blanksby, B.A., & Quek, K.L. (1999). Learning to swim using buoyancy aides. Pediatric Exercise Science, 11(4), 377-392.
5. Bautista, E.Q., Piqueras, J.A.S., Gonzalez, M.P.L., & Jordan, O.C. (2018). Influence of different aquatic materials on the perceived competence in the aquatic environment by students in the second cycle of early childhood education. Sport Tk-Euro-American Journal of Sports Sciences, 7(2), 73–79.

**Notes**

a. High risk of bias in the randomization process, high risk of bias due to missing outcome data, high risk of bias in the measurement of the outcome, and high risk of bias in the selection of results (Cochrane Risk of Bias-2).
b. Limited sample size.

c. High risk of bias in the randomization process, high risk of bias due to missing outcome data, high risk of bias in the measurement of the outcome, and some concerns on the selection of results (Cochrane Risk of Bias-2).

d. Moderate risk of bias due to confounding, moderate risk of bias due to missing data, and moderate risk of bias in the measurement of the outcome (ROBINS-I).

e. Downgraded by 2 levels for imprecision due to a limited sample size and a lack of data prohibiting us to assess whether the 95%CI contained both appreciable harm and benefit.

f. 95% confidence interval was not reported in the study and could not be calculated due to a lack of data.

g. Serious risk of bias due to confounding and moderate risk of bias in the measurement of the outcome (ROBINS-I).

h. Low number of events.

i. Downgraded by 2 levels for imprecision due to a limited sample size and a large variability of results.

## Comparison 1.9. An educational programme for basic swimming skills using goggles and snorkels vs an educational programme for basic swimming skills without the use of goggles and snorkels

| N° of studies | Study design | Risk of bias | Inconsistency | Indirectness | Imprecision | Other considerations | Number of participants | | Effect | | | Certainty |
| --- | --- | --- | --- | --- | --- | --- | --- | --- | --- | --- | --- | --- |
|  |  |  |  |  |  |  | Intervention | Control | Relative (95%CI) | Absolute (95%CI) | |  |
| Water safety skills: water entry test in children with no fear of water (Scale from 1 to 5), Δ post-pre | | | | | | | | | | | | |
| 1^1^ | Experimental: RCT | very serious^a^ | not serious | not serious | very serious^b^ | none | 16 | 18 | - | MD: 0.3 higher (0.25 lower to 0.85 higher) | 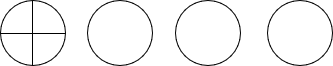 Very low | |
| Water safety skills: water entry test in children with high fear of water (Scale from 1 to 5), Δ post-pre | | | | | | | | | | | | |
| 1^2^ | Experimental: RCT | very serious^a^ | not serious | not serious | serious^c^ | none | 17 | 19 | - | MD: 0.9 higher (0.19 to 1.61 higher) | 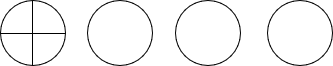 Very low | |
| Water safety skills: opening eyes underwater test in children with no fear of water (Scale from 1 to 2), Δ post-pre | | | | | | | | | | | | |
| 1^1^ | Experimental: RCT | very serious^a^ | not serious | not serious | very serious^b^ | none | 16 | 18 | - | MD: 0.2 higher (0.07 lower to 0.47 higher) | 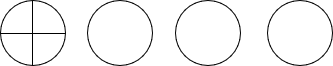 Very low | |
| Water safety skills: opening eyes underwater test in children with high fear of water (Scale from 1 to 2), Δ post-pre | | | | | | | | | | | | |
| 1^2^ | Experimental: RCT | very serious^a^ | not serious | not serious | very serious^b^ | none | 17 | 19 | - | MD: 0.1 lower (0.43 lower to 0.23 higher) | 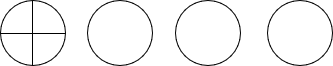 Very low | |
| Water safety skills: breath holding test in children with no fear of water (Scale from 1 to 5), Δ post-pre | | | | | | | | | | | | |
| 1^1^ | Experimental: RCT | very serious^a^ | not serious | not serious | serious^c^ | none | 16 | 18 | - | MD: 0.6 higher (0.21 lower to 1.41 higher) | 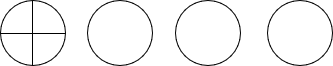 Very low | |
| Water safety skills: breath holding test in children with high fear of water (Scale from 1 to 5), Δ post-pre | | | | | | | | | | | | |
| 1^2^ | Experimental: RCT | very serious^a^ | not serious | not serious | serious^c^ | none | 17 | 19 | - | MD: 0.1 higher (0.65 lower to 0.85 higher) | 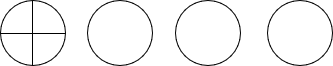 Very low | |
| Water safety skills: blowing bubbles test in children with no fear of water (Scale from 1 to 5), Δ post-pre | | | | | | | | | | | | |
| 1^1^ | Experimental: RCT | very serious^a^ | not serious | not serious | serious^c^ | none | 16 | 18 | - | MD: 1.1 lower (1.95 to 0.25 lower) | 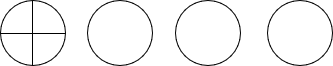 Very low | |
| Water safety skills: blowing bubbles test in children with high fear of water (Scale from 1 to 5), Δ post-pre | | | | | | | | | | | | |
| 1^2^ | Experimental: RCT | very serious^a^ | not serious | not serious | serious^c^ | none | 17 | 19 | - | MD: 0.5 lower (0.94 to 0.06 lower) | 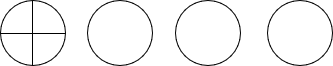 Very low | |
| Water safety skills: prone gliding test in children with no fear of water (Scale from 1 to 5), Δ post-pre | | | | | | | | | | | | |
| 1^1^ | Experimental: RCT | very serious^a^ | not serious | not serious | serious^c^ | none | 16 | 18 | - | MD: 0.5 lower (1.09 lower to 0.09 higher) | 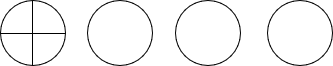 Very low | |
| Water safety skills: prone gliding test in children with high fear of water (Scale from 1 to 5), Δ post-pre | | | | | | | | | | | | |
| 1^2^ | Experimental: RCT | very serious^a^ | not serious | not serious | serious^c^ | none | 17 | 19 | - | MD: 0.4 higher (0.16 lower to 0.96 higher) | 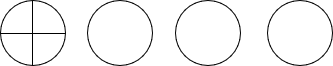 Very low | |
| Water safety skills: back gliding test in children with no fear of water (Scale from 1 to 5), Δ post-pre | | | | | | | | | | | | |
| 1^1^ | Experimental: RCT | very serious^a^ | not serious | not serious | serious^c^ | none | 16 | 18 | - | MD: 0.7 lower (1.44 lower to 0.04 higher) | 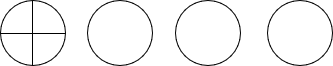 Very low | |
| Water safety skills: back gliding test in children with high fear of water (Scale from 1 to 5), Δ post-pre | | | | | | | | | | | | |
| 1^2^ | Experimental: RCT | very serious^a^ | not serious | not serious | serious^c^ | none | 17 | 19 | - | MD: 0.8 higher (0.15 to 1.45 higher) | 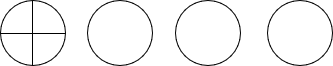 Very low | |
| Water safety skills: front-back-front roll test in children with no fear of water (Scale from 1 to 5), Δ post-pre | | | | | | | | | | | | |
| 1^1^ | Experimental: RCT | very serious^a^ | not serious | not serious | serious^c^ | none | 16 | 18 | - | MD: 0.3 higher (0.37 lower to 0.97 higher) | 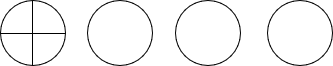 Very low | |
| Water safety skills: front-back-front roll test in children with high fear of water (Scale from 1 to 5), Δ post-pre | | | | | | | | | | | | |
| 1^2^ | Experimental: RCT | very serious^a^ | not serious | not serious | serious^c^ | none | 17 | 19 | - | MD: 0.6 higher (0.05 lower to 1.25 higher) | 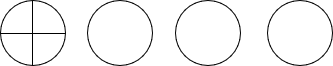 Very low | |
| Water safety skills: horizontal changing position test in children with no fear of water (Scale from 1 to 5), Δ post-pre | | | | | | | | | | | | |
| 1^1^ | Experimental: RCT | very serious^a^ | not serious | not serious | serious^c^ | none | 16 | 18 | - | MD: 0.00 (0.87 lower to 0.87 higher) | 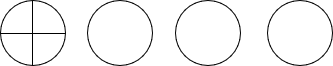 Very low | |
| Water safety skills: horizontal changing position test in children with high fear of water (Scale from 1 to 5), Δ post-pre | | | | | | | | | | | | |
| 1^2^ | Experimental: RCT | very serious^a^ | not serious | not serious | serious^c^ | none | 17 | 19 | - | MD: 0.4 higher (0.45 lower to 1.25 higher) | 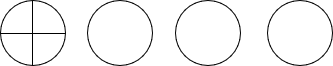 Very low | |
| Water safety skills: prone swim test in children with no fear of water (Scale from 1 to 5), Δ post-pre | | | | | | | | | | | | |
| 1^1^ | Experimental: RCT | very serious^a^ | not serious | not serious | serious^c^ | none | 16 | 18 | - | MD: 1.5 higher (0.48 to 2.52 higher) | 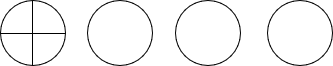Very low | |
| Water safety skills: prone swim test in children with high fear of water (Scale from 1 to 5), Δ post-pre | | | | | | | | | | | | |
| 1^2^ | Experimental: RCT | very serious^a^ | not serious | not serious | serious^c^ | none | 17 | 19 | - | MD: 0.7 higher (0.03 to 1.37 higher) | 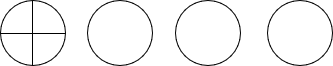 Very low | |
| Water safety skills: breathing during prone swim test in children with no fear of water (Scale from 1 to 5), Δ post-pre | | | | | | | | | | | | |
| 1^1^ | Experimental: RCT | very serious^a^ | not serious | not serious | serious^c^ | none | 16 | 18 | - | MD: 0.6 lower (1.16 to 0.04 lower) | 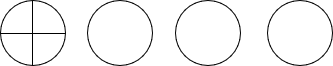 Very low | |
| Water safety skills: breathing during prone swim test in children with high fear of water (Scale from 1 to 5), Δ post-pre | | | | | | | | | | | | |
| 1^2^ | Experimental: RCT | very serious^a^ | not serious | not serious | serious^c^ | none | 17 | 19 | - | MD: 0.8 lower (1.62 lower to 0.02 higher) | 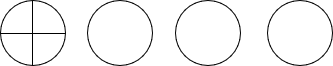 Very low | |
| Water safety skills: back swim test in children with no fear of water (Scale from 1 to 5), Δ post-pre | | | | | | | | | | | | |
| 1^1^ | Experimental: RCT | very serious^a^ | not serious | not serious | serious^c^ | none | 16 | 18 | - | MD: 0.2 lower (1.21 lower to 0.81 higher) | 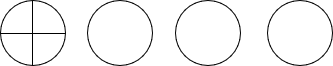 Very low | |
| Water safety skills: back swim test in children with high fear of water (Scale from 1 to 5), Δ post-pre | | | | | | | | | | | | |
| 1^2^ | Experimental: RCT | very serious^a^ | not serious | not serious | serious^c^ | none | 17 | 19 | - | MD: 0.4 higher (0.32 lower to 1.12 higher) | 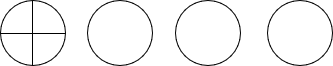 Very low | |

**References**

1. Misimi, F., Kajtna, T., Stirn, I., Zoretic, D., Misimi, S., & Kapus, J. (2023). More on the use of goggles and snorkel in learning-to-swim: new results for children without fear of water. Perceptual and Motor Skills, 13(4), 1714-1731.
2. Misimi, F., Kajtna, T., & Kapus, J. (2022). The effect of using goggles and snorkel for aquatic skills acquisition in youth learn-to-swim programs. Perceptual and Motor Skills, 129(5), 1525-1545.

**Notes**

a. High risk of bias in the ranomization process, some concerns on the selection of results (Cochrane Risk of Bias-2).
b. Downgraded by 2 levels for imprecision due to a limited sample size and a large variability of results.
c. Limited sample size.

## Comparison 1.10. An educational programme for basic swimming skills using video-taped feedback vs an educational programme for basic swimming skills using auditory feedback

| N° of studies | Study design | Risk of bias | Inconsistency | | Indirectness | Imprecision | Other considerations | Number of participants | | Effect | | Certainty |
| --- | --- | --- | --- | --- | --- | --- | --- | --- | --- | --- | --- | --- |
|  |  |  |  |  |  |  |  | Intervention | Control | Relative (95%CI) | Absolute (95%CI) |  |
| Water safety skills: flutter kick performance in children aged 4.5 to 6.4 years (Scale from 1 to 6), at the end of the intervention programme | | | | | | | | | | | | |
| 1^1^ | Experimental: RCT | serious^a^ | | not serious | not serious | serious^b^ | none | 9 | 9 | - | MD: 0.38 higher (-- to --)^c^ (p > 0.05) | 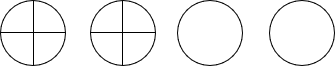 Low |
| Water safety skills: flutter kick performance in children aged 6.5 to 8.5 years (Scale from 1 to 6), at the end of the intervention programme | | | | | | | | | | | | |
| 1^1^ | Experimental: RCT | serious^a^ | | not serious | not serious | serious^b^ | none | 9 | 9 | - | MD: 0.73 higher (-- to --)^c^ (p < 0.05) | 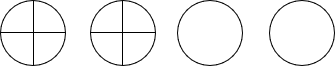 Low |

**References:**

1. Bunker, L.K., Shearer, J.D., & Hall, E.G. (1976). Video-taped feedback and children's learning to flutter kick. Perceptual and Motor Skills, 43, 371-374.

**Notes**

a. Some concerns on the randomization process and some concerns on the selection of the reported results (Cochrane Risk of Bias-2).
b. Limited sample size.
c. 95% confidence interval was not reported in the study and could not be calculated due to multiple comparisons.

## Comparison 1.11. An educational programme for basic swimming skills in shallow water vs an educational programme for basic swimming skills in deep water

| N° of studies | Study design | Risk of bias | Inconsistency | | Indirectness | | Imprecision | | Other considerations | | Number of participants | | | | Effect | | | Certainty |
| --- | --- | --- | --- | --- | --- | --- | --- | --- | --- | --- | --- | --- | --- | --- | --- | --- | --- | --- |
|  |  |  |  |  |  |  |  |  |  |  | Intervention | | Control | | Relative (95%CI) | Absolute (95%CI) | |  |
| Water safety skills: aquatic skills in domain "water entry" (Scale from 1 to 3), at the end of the intervention programme | | | | | | | | | | | | | | | | | | |
| 1^1^ | Observational: cohort study | serious^a^ | | not serious | | not serious | | serious^b^ | | none | | 16 | | 16 | - | | MD: 0.37 higher (not estimable)  (p < 0.05)^c^ | 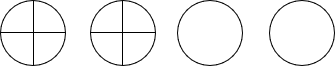 Low |
| 1^2^ | Experimental: non-RCT | very serious^d^ | | not serious | | not serious | | serious^b^ | | none | | 11 | | 10 | - | | MD: 0.10 (not estimable)  (p > 0.05)^e^ | 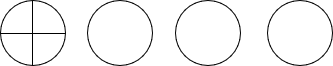 Very low |
| Water safety skills: aquatic skills in domain "water orientation and adjustment at vertical position" (Scale from 1 to 3), at the end of the intervention programme | | | | | | | | | | | | | | | | | | |
| 1^1^ | Observational: cohort study | serious^a^ | | not serious | | not serious | | serious^b^ | | none | | 16 | | 16 | - | | MD: 0.25 higher (0.05 lower to 0.55 higher) | 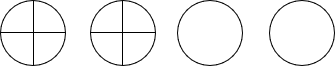 Low |
| 1^2^ | Experimental: non-RCT | very serious^d^ | | not serious | | not serious | | serious^b^ | | none | | 11 | | 10 | - | | MD: 3.00 higher (not estimable) | 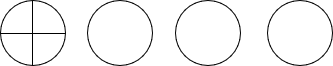 Very low |
| Water safety skills: aquatic skills in domain "breath control" (Scale from 1 to 5), at the end of the intervention programme | | | | | | | | | | | | | | | | | | |
| 1^1^ | Observational: cohort study | serious^a^ | | not serious | | not serious | | serious^b^ | | none | | 16 | | 16 | - | | MD: 0.32 higher (0.31 lower to 0.95 higher) | 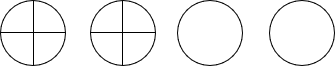 Low |
| 1^2^ | Experimental: non-RCT | very serious^d^ | | not serious | | not serious | | serious^b^ | | none | | 11 | | 10 | - | | MD: 1.09 higher (0.56 to 1.62 higher) | 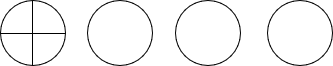 Very low |
| Water safety skills: aquatic skills in domain "horizontal buoyancy" (Scale from 1 to 4), at the end of the intervention programme | | | | | | | | | | | | | | | | | | |
| 1^1^ | Observational: cohort study | serious^a^ | | not serious | | not serious | | serious^b^ | | none | | 16 | | 16 | - | | MD: 1.75 higher (1.13 to 2.37 higher) | 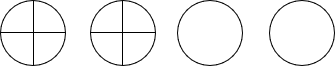 Low |
| 1^2^ | Experimental: non-RCT | very serious^d^ | | not serious | | not serious | | serious^b^ | | none | | 11 | | 10 | - | | MD: 1.14 higher (0.60 to 1.67 higher) | 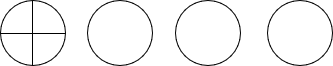 Very low |
| Water safety skills: aquatic skills in domain "body position at vertical gliding" (Scale from 1 to 4), at the end of the intervention programme | | | | | | | | | | | | | | | | | | |
| 1^1^ | Observational: cohort study | serious^a^ | | not serious | | not serious | | serious^b^ | | none | | 16 | | 16 | - | | MD: 2.00 higher (1.41 to 2.59 higher) | 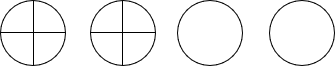Low |
| 1^2^ | Experimental: non-RCT | very serious^d^ | | not serious | | not serious | | serious^b^ | | none | | 11 | | 10 | - | | MD: 1.53 higher (1.07 to 1.99 higher) | 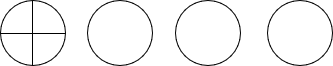 Very low |
| Water safety skills: aquatic skills in domain "body position at dorsal gliding" (Scale from 1 to 4), at the end of the intervention programme | | | | | | | | | | | | | | | | | | |
| 1^1^ | Observational: cohort study | serious^a^ | | not serious | | not serious | | serious^b^ | | none | | 16 | | 16 | - | | MD: 1.88 higher (not estimable)  (p < 0.05)^c^ | 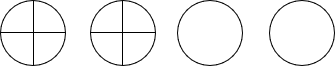 Low |
| 1^2^ | Experimental: non-RCT | very serious^d^ | | not serious | | not serious | | serious^b^ | | none | | 11 | | 10 | - | | MD: 0.99 higher (0.46 to 1.52 higher) | 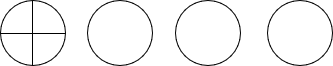 Very low |
| Water safety skills: aquatic skills in domain "body position at longitudinal rotation in gliding" (Scale from 1 to 3), at the end of the intervention programme | | | | | | | | | | | | | | | | | | |
| 1^1^ | Observational: cohort study | serious^a^ | | not serious | | not serious | | serious^b^ | | none | | 16 | | 16 | - | | MD: 0.88 higher (0.58 to 1.18 higher) | 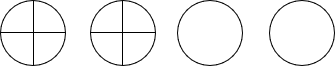 Low |
| 1^2^ | Experimental: non-RCT | very serious^d^ | | not serious | | not serious | | serious^b^ | | none | | 11 | | 10 | - | | MD: 0.16 higher (0.27 lower to 0.59 higher) | 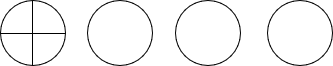 Very low |
| Water safety skills: aquatic skills in domain "body position at front and back somersaults" (Scale from 1 to 4), at the end of the intervention programme | | | | | | | | | | | | | | | | | | |
| 1^1^ | Observational: cohort study | serious^a^ | | not serious | | not serious | | serious^b^ | | none | | 16 | | 16 | - | | MD: 0.88 higher (0.26 to 1.50 higher) | 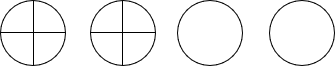 Low |
| 1^2^ | Experimental: non-RCT | very serious^d^ | | not serious | | not serious | | serious^b^ | | none | | 11 | | 10 | - | | MD: 0.00 higher (not estimable) | 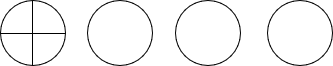 Very low |
| Water safety skills: aquatic skills in domain "leg kick with breath control at ventral position with flutter boards" (Scale from 1 to 4), at the end of the intervention programme | | | | | | | | | | | | | | | | | | |
| 1^1^ | Observational: cohort study | serious^a^ | | not serious | | not serious | | serious^b^ | | none | | 16 | | 16 | - | | MD: 1.44 higher (0.94 to 1.94 higher) | Low |
| 1^2^ | Experimental: non-RCT | very serious^d^ | | not serious | | not serious | | serious^b^ | | none | | 11 | | 10 | - | | MD: 0.36 higher (0.01 lower to 0.73 higher) | Very low |
| Water safety skills: aquatic skills in domain "leg kick with breath control at ventral body position without any flutter device" (Scale from 1 to 4), at the end of the intervention programme | | | | | | | | | | | | | | | | | | |
| 1^1^ | Observational: cohort study | serious^a^ | | not serious | | not serious | | serious^b^ | | none | | 16 | | 16 | - | | MD: 1.32 higher (0.86 to 1.78 higher) | Low |
| 1^2^ | Experimental: non-RCT | very serious^d^ | | not serious | | not serious | | serious^b^ | | none | | 11 | | 10 | - | | MD: 0.60 higher (0.11 to 1.09 higher) | Very low |
| Water safety skills: aquatic skills in domain "leg kick with breath control at dorsal body position with flutter boards" (Scale from 1 to 4), at the end of the intervention programme | | | | | | | | | | | | | | | | | | |
| 1^1^ | Observational: cohort study | serious^a^ | | not serious | | not serious | | serious^b^ | | none | | 16 | | 16 | - | | MD: 1.13 higher (0.75 to 1.51 higher) | Low |
| 1^2^ | Experimental: non-RCT | very serious^d^ | | not serious | | not serious | | serious^b^ | | none | | 11 | | 10 | - | | MD: 0.39 higher (0.20 lower to 0.98 higher) | Very low |
| Water safety skills: aquatic skills in domain "leg kick with breath control at dorsal body position without any flutter device" (Scale from 1 to 4), at the end of the intervention programme | | | | | | | | | | | | | | | | | | |
| 1^1^ | Observational: cohort study | serious^a^ | | not serious | | not serious | | serious^b^ | | none | | 16 | | 16 | - | | MD: 1.19 higher (0.79 to 1.59 higher) | Low |
| 1^2^ | Experimental: non-RCT | very serious^d^ | | not serious | | not serious | | serious^b^ | | none | | 11 | | 10 | - | | MD: 0.62 higher (-- to --)^f^ (p > 0.05) | Very low |
| Water safety skills: aquatic skills in domain "feet-first entry" (Scale from 1 to 3), at the end of the intervention programme | | | | | | | | | | | | | | | | | | |
| 1^1^ | Observational: cohort study | serious^a^ | | not serious | | not serious | | serious^b^ | | none | | 16 | | 16 | - | | MD: 0.56 higher (0.14 to 0.98 higher) | Low |
| 1^2^ | Experimental: non-RCT | very serious^d^ | | not serious | | not serious | | serious^b^ | | none | | 11 | | 10 | - | | MD: 0.55 higher (0.02 lower to 1.12 higher) | Very low |
| Water safety skills: aquatic skills in domain "head-first entry" (Scale from 1 to 3), at the end of the intervention programme | | | | | | | | | | | | | | | | | | |
| 1^1^ | Observational: cohort study | serious^a^ | | not serious | | not serious | | serious^b^ | | none | | 16 | | 16 | - | | MD: 0.75 higher (0.29 to 1.21 higher) | Low |
| 1^2^ | Experimental: non-RCT | very serious^d^ | | not serious | | not serious | | serious^b^ | | none | | 11 | | 10 | - | | MD: 0.43 higher (0.07 lower to 0.93 higher) | Very low |
| Water safety skills: aquatic skills in domain "autonomous in deep pool, legs and arms displacement" (Scale from 1 to 3), at the end of the intervention programme | | | | | | | | | | | | | | | | | | |
| 1^1^ | Observational: cohort study | serious^a^ | | not serious | | not serious | | serious^b^ | | none | | 16 | | 16 | - | | MD: 0.44 higher (0.09 lower to 0.97 higher) | Low |
| 1^2^ | Experimental: non-RCT | very serious^d^ | | not serious | | not serious | | serious^b^ | | none | | 11 | | 10 | - | | MD: 0.14 lower (0.59 lower to 0.31 higher) | Very low |
| Water safety skills: aquatic skills in domain "vertical buoyancy at deep water" (Scale from 1 to 5), at the end of the intervention programme | | | | | | | | | | | | | | | | | | |
| 1^1^ | Observational: cohort study | serious^a^ | | not serious | | not serious | | serious^b^ | | none | | 16 | | 16 | - | | MD: 1.62 higher (0.76 to 2.48 higher) | Low |
| 1^2^ | Experimental: non-RCT | very serious^d^ | | not serious | | not serious | | serious^b^ | | none | | 11 | | 10 | - | | MD: 0.34 lower (0.76 lower to 0.08 higher) | Very low |
| Water safety skills: aquatic skills in domain "deep-water immersion" (Scale from 1 to 4), at the end of the intervention programme | | | | | | | | | | | | | | | | | | |
| 1^1^ | Observational: cohort study | serious^a^ | | not serious | | not serious | | serious^b^ | | none | | 16 | | 16 | - | | MD: 1.37 higher (0.69 to 2.05 higher) | Low |
| 1^2^ | Experimental: non-RCT | very serious^d^ | | not serious | | not serious | | serious^b^ | | none | | 11 | | 10 | - | | MD: 0.12 lower (0.50 lower to 0.26 higher) | Very low |

**References**

1. Costa, A.M., Marinho, D.A., Rocha, H., Silva, A.J., Barbosa, T.M., Ferreira, S.S., & Martins, M. (2012). Deep and shallow water effects on developing preschoolers’ aquatic skills. Journal of Human Kinetics, 32, 211–219.
2. Rocha, H.A., Marinho, D.A., Garrido, N.D., Morgado, L.S., & Costa, A.M. (2018). The acquisition of aquatic skills in preschool children: deep versus shallow water swimming lessons. Motricidade, 14(1), 66-72.

**Notes**

a. Moderate risk of bias due to confounding, and moderate risk of bias in the measurement of the outcome (ROBINS-I).
b. Limited sample size.
c. 95% confidence interval was not reported in the study and was not estimable using Review Manager. Reported p-value results from a Fisher's exact analysis executed by the study authors.
d. Serious risk of bias due to confounding, and moderate risk of bias in the measurement of the outcome (ROBINS-I).
e. 95% confidence interval was not reported in the study and was not estimable using Review Manager. Reported p-value results from a Student's t-test executed by the study authors.

f. 95%CI not reported due to a discrepancy between p value reported by study authors (p=0.057) and 95%CI calculated in Review Manager.

# Part 2 - Water safety training (out-of-water)

## Comparison 2.1. An educational programme for water safety skills without in-water training vs no educational programme for water safety skills

| N° of studies | | Study design | Risk of bias | Inconsistency | Indirectness | Imprecision | Other considerations | Number of participants | | Effect | | Certainty |
| --- | --- | --- | --- | --- | --- | --- | --- | --- | --- | --- | --- | --- |
|  |  |  |  |  |  |  |  | Intervention | Control | Relative (95%CI) | Absolute (95%CI) |  |
| Drowning-related mortality | | | | | | | | | | | | |
| 1^1^ | Observational: case-control study | | serious^a^ | not serious | not serious | serious^b^ | none | 79 cases, 79 controls | | aOR: 0.23  (0.07 to 0.74)^c^ | - | Low |
| Water safety knowledge: Safety knowledge on drowning risks (%), 1 week after intervention | | | | | | | | | | | | |
| 1^2^ | Experimental: RCT | | not serious | not serious | not serious | not serious | none | 137 | 143 | - | MD: 3.61 higher (-- to --)^d^ (p < 0.05) | High |
| Water safety knowledge: Drowning prevention knowledge (/20), at the end of the intervention programme | | | | | | | | | | | | |
| 1^3^ | Experimental: non-RCT | | serious^e^ | not serious | not serious | not serious | none | 60 | 60 | - | MD: 3.00 higher (2.07 to 3.39 higher) | Moderate |
| Water safety knowledge (%) in children aged 5-6 years, 1 month after intervention | | | | | | | | | | | | |
| 1^4^ | Experimental: non-RCT | | very serious^f^ | not serious | not serious | serious^b^ | none | 115 | 202 | - | aMD: 17.40 higher (6.41 to 28.39 higher) | Very low |
| Water safety knowledge (%) in children aged 7-11 years, 1 month after intervention | | | | | | | | | | | | |
| 1^4^ | Experimental: non-RCT | | very serious^f^ | not serious | not serious | serious^b^ | none | 205 | 220 | - | aMD: 14.58 higher (3.05 lower to 32.21 higher) | Very low |
| Water safety knowledge (%), in children aged 12-15 years, 1 month after intervention | | | | | | | | | | | | |
| 1^4^ | Experimental: non-RCT | | very serious^f^ | not serious | not serious | serious^b^ | none | 321 | 337 | - | aMD: 0.15 lower (5.30 lower to 4.99 higher) | Very low |
| Water safety knowledge: Knowledge of prevention of drowning on the beach (%), at the end of the intervention programme | | | | | | | | | | | | |
| 1^5^ | Experimental: non-RCT | | very serious^g^ | not serious | not serious | serious^b^ | none | 14 | 12 | - | MD: 53.28 higher (36.05 to 70.51 higher) | Very low |
| Water safety knowledge: Knowledge of prevention of drowning on the beach (%), 1 month after the intervention | | | | | | | | | | | | |
| 1^5^ | Experimental: non-RCT | | very serious^g^ | not serious | not serious | serious^b^ | none | 14 | 12 | - | MD: 49.71 higher (37.39 to 62.03 higher) | Very low |
| Water safety knowledge: Knowledge of prevention of drowning in the swimming pool (%), at the end of the intervention programme | | | | | | | | | | | | |
| 1^5^ | Experimental: non-RCT | | very serious^g^ | not serious | not serious | serious^b^ | none | 14 | 12 | - | MD: 56.67 higher (38.80 to 74.54 higher) | Very low |
| Water safety knowledge: Knowledge of prevention of drowning in the swimming pool (%), 1 month after the intervention | | | | | | | | | | | | |
| 1^5^ | Experimental: non-RCT | | very serious^g^ | not serious | not serious | serious^b^ | none | 14 | 12 | - | MD: 33.10 higher (17.57 to 48.63 higher) | Very low |
| Water safety knowledge: Overall knowledge on prevention of drowning (%), at the end of the intervention programme | | | | | | | | | | | | |
| 1^5^ | Experimental: non-RCT | | very serious^g^ | not serious | not serious | serious^b^ | none | 14 | 12 | - | MD: 54.97 higher (41.85 to 68.09 higher) | Very low |
| Water safety knowledge: Overall knowledge on prevention of drowning (%), 1 month after the intervention | | | | | | | | | | | | |
| 1^5^ | Experimental: non-RCT | | very serious^g^ | not serious | not serious | serious^b^ | none | 14 | 12 | - | MD: 41.40 higher (29.90 to 52.90 higher) | Very low |
| Water safety behaviour: Perceived vulnerability toward drowning risk (Scale from 0 to 5), 1 week after the intervention | | | | | | | | | | | | |
| 1^2^ | Experimental: RCT | | not serious | not serious | not serious | not serious | none | 137 | 143 | - | MD: 0.01 lower (-- to --)^d^ (p > 0.05) | High |
| Water safety behaviour: Simulated behaviour around water (Scale from 0 to 1, higher score indicates more risky behaviour), 1 week after the intervention | | | | | | | | | | | | |
| 1^2^ | Experimental: RCT | | not serious | not serious | not serious | not serious | none | 135 | 141 | - | MD: 0.04 lower (-- to --)^d^ (p < 0.05) | High |
| Water safety behaviour: Drowning prevention attitudes (/60), at the end of the intervention programme | | | | | | | | | | | | |
| 1^3^ | Experimental: non-RCT | | very serious^h^ | not serious | not serious | not serious | none | 60 | 60 | - | MD: 6.86 higher (5.37 to 8.35 higher) | Low |
| Water safety behaviour: Drowning prevention behaviour, at the end of the intervention programme | | | | | | | | | | | | |
| 1^3^ | Experimental: non-RCT | | very serious^h^ | not serious | not serious | not serious | none | 60 | 60 | - | MD: 3.15 higher (2.23 to 4.07 higher) | Low |
| Water safety behaviour: attitudes on water safety (%) in children aged 5-6 years, 1 month after the intervention | | | | | | | | | | | | |
| 1^4^ | Experimental: non-RCT | | very serious^f^ | not serious | not serious | serious^b^ | none | 115 | 202 | - | aMD: 23.64 higher (4.48 to 42.79 higher) | Very low |
| Water safety behaviour: attitudes on water safety (%) in children aged 7-11 years, 1 month after the intervention | | | | | | | | | | | | |
| 1^4^ | Experimental: non-RCT | | very serious^f^ | not serious | not serious | very serious^i^ | none | 205 | 220 | - | aMD: 5.64 higher (11.47 lower to 22.77 higher) | Very low |
| Water safety behaviour: attitudes on water safety (%) in children aged 12-15 years, 1 month after the intervention | | | | | | | | | | | | |
| 1^4^ | Experimental: non-RCT | | very serious^f^ | not serious | not serious | serious^b^ | none | 321 | 337 | - | aMD: 6.32 higher (1.87 lower to 14.52 higher) | Very low |

**References**

1. Liu, Z., Kong, F., Yin, L., Wang, A., Xiong, L., & Xie, D. (2019). Epidemiological characteristics and influencing factors of fatal drowning in children under 5 years old in Hunan Province, China: case-control study. BMC Public Health, 19(1), 955.
2. Shen, J,. Pang, S., & Schwebel, D.C. (2016). Evaluation of a drowning prevention program based on testimonial videos: a randomized controlled trial. Journal of Pediatric Psychology, 41(5), 555–565.
3. Kusol, K., Phromphen, C., & Eksirinimit, T. (2020). Effects of potential support program on drowning prevention among primary school students in Nakhon Si Thammarat Province, Thailand. Sustainability, 14, 11717.
4. Terzidis, A., Koutroumpa, A., Skalkidis, I., Matzavakis, I., Malliori, M., & Frangakis, C.E. (2007). Water safety: age-specific changes in knowledge and attitudes following a school-based intervention. Injury Prevention, 13(2), 120–124.
5. Barcala-Furelos, R., Carbia-Rodriguez, P., Peixoto-Pino, L., Abelairas-Gomez, C., & Rodriguez-Nunez, A. (2019). Implementation of educational programs to prevent drowning. What can be done in nursery school? Medicina Intensiva, 43(3), 180–182.

**Notes**

a. Moderate risk of bias due to confounding, moderate risk of bias in the classification of interventions (potential recall bias), and moderate risk of bias due to missing data (ROBINS-I).
b. Limited sample size.
c. The authors only reported a 90% CI. This CI therefore represents the 90% CI and not the 95% CI.
d. 95% confidence interval was not reported in the study and could not be calculated due to multiple comparisons.
e. Moderate risk of bias due to confounding (ROBINS-I).
f. Serious risk of bias in the measurement of the outcome, and moderate risk of bias due to confounding (ROBINS-I).
g. Serious risk of bias due to confounding (ROBINS-I).
h. Serious risk of bias in the measurement of the outcome, and moderate risk of bias due to confounding (ROBINS-I).

i. Downgraded by 2 levels due to the limited sample size and a large variability of results.

## Comparison 2.2. An educational programme for water safety skills without in-water training using virtual reality vs an educational programme for water safety skills using another mode of delivery

| N° of studies | Study design | Risk of bias | Inconsistency | Indirectness | | Imprecision | Other considerations | Number of participants | | Effect | | Certainty |
| --- | --- | --- | --- | --- | --- | --- | --- | --- | --- | --- | --- | --- |
|  |  |  |  |  |  |  |  | Intervention | Control | Relative (95%CI) | Absolute (95%CI) |  |
| Water safety knowledge | | | | | | | | | | | | |
| 1^1^ | Experimental: RCT | very serious^a^ | not serious | | not serious | very serious^b^ | none | This study compared delivery modes, virtual reality (n=46) vs traditional video (n=28) vs poster (n=41), with each other at three time points, immediately after training, 1 week after training and 8 weeks after training. p=0.123 in the repeated measures ANOVA analysis over the 3 intervention groups across the three time points | | | | Very low |

**Reference:**

1. Araiza-Alba, P., Kaene, T., Matthews, B., Simpson, K., Strugnell, G., Chen, W.S., & Kaufman, J. (2021). The potential of 360-degree virtual reality videos to teach water-safety skills to children. Computers & Education, 163, 104096.

**Notes**

a. High risk of bias due to missing outcome data, some concerns on the randomization process, and some concerns on the selection of the reported results (Cochrane Risk of Bias-2).
b. Downgraded by 2 levels due to the limited sample size and the lack of data, prohibiting us to assess whether the 95%CI contained both appreciable harm and benefit.

# Part 3 - Water safety training (out-of-water) as part of a broad educational programme on injury prevention

## Comparison 3.1. A broad educational programme containing out-of-water water safety skills training vs no broad educational programme for water safety skills training

| N° of studies | Study design | Risk of bias | Inconsistency | Indirectness | Imprecision | | Other considerations | | Number of participants | | | Effect | | | Certainty |
| --- | --- | --- | --- | --- | --- | --- | --- | --- | --- | --- | --- | --- | --- | --- | --- |
|  |  |  |  |  |  |  |  |  | Intervention | | Control | Relative (95%CI) | Absolute (95%CI) | |  |
| Water safety knowledge: Knowledge on good swimming habits | | | | | | | | | | | | | | | |
| 1^1^ | Experimental:  non-RCT | very serious^a^ | not serious | not serious | serious^b^ | none | | --/--^c^ | | --/--^c^ | | χ2: 17.6 (-- to --) (p < 0.01)^d^ | - | Very low | |
| Water safety knowledge: Knowledge on water safety rules | | | | | | | | | | | | | | | |
| 1^1^ | Experimental:  non-RCT | very serious^a^ | not serious | not serious | serious^b^ | none | | --/--^c^ | | --/--^c^ | | χ2: 9.9 (-- to --) (p < 0.01)^d^ | - | Very low | |
| Water safety knowledge | | | | | | | | | | | | | | | |
| 1^2^ | Experimental:  non-RCT | serious^e^ | not serious | not serious | serious^f^ | none | | 542 | | 554 | | - | MD: 0.00 (0.12 lower to 0.12 higher) | Low | |
| Water safety knowledge (change score, %), children in grade 1 of elementary school | | | | | | | | | | | | | | | |
| 1^3^ | Experimental:  non-RCT | serious^g^ | not serious | not serious | serious^b^ | none | | --^g^ | | -- ^g^ | | - | MD: 18.7 higher (-- to --)^i^ (p < 0.05) | Low | |
| Water safety knowledge (change score, %), children in grade 2 of elementary school | | | | | | | | | | | | | | | |
| 1^3^ | Experimental:  non-RCT | serious^g^ | not serious | not serious | serious^b^ | none | | -- ^g^ | | -- ^g^ | | - | MD: 22.2 higher (-- to --)^i^ (p < 0.05) | Low | |
| Water safety knowledge (change score, %), children in grade 3 of elementary school | | | | | | | | | | | | | | | |
| 1^3^ | Experimental:  non-RCT | serious^g^ | not serious | not serious | serious^b^ | none | | -- ^g^ | | -- ^g^ | | - | MD: 12.8 higher (-- to --)^i^ (p < 0.05) | Low | |
| Water safety behaviour: diving safety attitude | | | | | | | | | | | | | | | |
| 1^1^ | Experimental:  non-RCT | very serious^a^ | not serious | not serious | serious^b^ | none | | --/--^c^ | | --/--^c^ | | χ2: 7.1 (-- to --) (p < 0.01)^d^ | - | Very low | |
| Water safety behaviour: Being able to identify the risk "Toddler fall in water" | | | | | | | | | | | | | | | |
| 1^2^ | Experimental:  non-RCT | serious^e^ | not serious | not serious | serious^j^ | none | | 58/564 (10.3%) | | 34/550 (6.1%) | | RR: 1.69 (1.13 to 2.54) | - | Low | |
| Water safety behaviour: Being able to identify the risk "Playing with a ball near water" | | | | | | | | | | | | | | | |
| 1^2^ | Experimental:  non-RCT | serious^e^ | not serious | not serious | serious^j^ | none | | 260/564 (46.1%) | | 314/560 (56.1%) | | RR: 0.82 (0.73 to 0.92) | - | Low | |
| Water safety behaviour: Being able to identify the general water danger | | | | | | | | | | | | | | | |
| 1^2^ | Experimental:  non-RCT | serious^e^ | not serious | not serious | serious^j^ | none | | 58/564 (10.3%) | | 82/560 (14.6%) | | RR: 0.70 (0.51 to 0.96) | - | Low | |
| Water safety behaviour: Being able to decide to stop playing near water | | | | | | | | | | | | | | | |
| 1^2^ | Experimental:  non-RCT | serious^e^ | not serious | not serious | serious^j^ | none | | 141/564 (25.0%) | | 98/550 (17.8%) | | RR: 1.40 (1.12 to 1.76) | - | Low | |
| Water safety behaviour: Being able to decide not to play near water | | | | | | | | | | | | | | | |
| 1^2^ | Experimental:  non-RCT | serious^e^ | not serious | not serious | serious^j^ | none | | 58/564 (10.3%) | | 89/550 (16.2%) | | RR: 0.64 (0.47 to 0.87) | - | Low | |
| Water safety behaviour: Completely or partially agreeing with "Will check the depth of the swimming pool", at the end of the intervention | | | | | | | | | | | | | | | |
| 1^4^ | Experimental:  (cluster)-RCT | very serious^k^ | not serious | not serious | not serious | none | | 559/572 (97.7%) | | 442/477 (92.6%) | | RR: 1.05 (1.03 to 1.08) | - | Low | |
| Water safety behaviour: Completely or partially agreeing with "Will check the depth of the swimming pool", at 5 months follow-up | | | | | | | | | | | | | | | |
| 1^4^ | Experimental:  (cluster-)RCT | very serious^k^ | not serious | not serious | not serious | none | | 531/572 (92.8%) | | 429/477 (89.9%) | | RR: 1.03 (0.99 to 1.07) | - | Low | |

**References**

1. Azeredo, R., & Stephens-Stidham, S. (2003). Design and implementation of injury prevention curricula for elementary schools: lessons learned. Injury Prevention, 9(3), 274–278.
2. Frederick, K., Bixby, E., Orzel, M.N., Stewart-Brown, S., & Willett, K. (2000). An evaluation of the effectiveness of the Injury Minimization Programme for Schools (IMPS). Injury Prevention, 6(2), 92–95.
3. Greene, A., Barnett, P., Crossen, J., Sexton, G., Ruzicka, P., & Neuwelt, E. (2002). Evaluation of the THINK FIRST For KIDS injury prevention curriculum for primary students. Injury Prevention, 8(3), 257–258.
4. Falavigna, A., Teles, A.R., Velho, M.C., Medeiros, G.S., Canabarro, C.T., de Braga, G.L., ... & Kleber, F.D. (2012). Impact of an injury prevention program on teenagers’ knowledge and attitudes: results of the Pense Bem-Caxias do Sul Project. Journal of Neurosurgery: Pediatrics, 9(5), 562–568.

**Notes**

a. Serious risk of bias due to confounding (ROBINS-I).
b. Lack of data, prohibiting us to assess whether the 95%CI contained both appreciable harm and benefit
c. Number of completed surveys not available per group.
d. 95% confidence interval was not reported in the study and could not be calculated due to a lack of data. P values as reported by the study authors.
e. Moderate risk of bias due to confounding, and moderate risk of bias due to missing data (ROBINS-I).
f. Limited sample size.
g. Moderate risk of bias due to confounding (ROBINS-I).
h. Number of students not available per grade.
o. 95% confidence interval was not reported in the study and could not be calculated due to a lack of data.
j. Low number of events.
k. High risk of bias in the measurement of the outcome, some concerns on the randomization process and some concerns on the selection of the reported results (Cochrane Risk of Bias-2).

## Comparison 3.2. A multimodal educational programme for water safety skills training (out-of-water) vs handbook education only

| N° of studies | Study design | Risk of bias | Inconsistency | Indirectness | Imprecision | | Other considerations | | Number of participants | | | Effect | | | Certainty |
| --- | --- | --- | --- | --- | --- | --- | --- | --- | --- | --- | --- | --- | --- | --- | --- |
|  |  |  |  |  |  |  |  |  | Intervention | | Control | Relative (95%CI) | Absolute (95%CI) | |  |
| Water safety knowledge: Knowledge on good swimming habits, 16 months after the intervention | | | | | | | | | | | | | | | |
| 1^1^ | Experimental:  RCT | very serious^a^ | not serious | not serious | not serious | none | | 841 | | 661 | | - | MD: 0.05 higher (0.1 lower to 0.2 higher) | Low | |

**References**

1. Cao, B.L., Shi, X.Q., Qi, Y.H., Hui, Y., Yang, H.J., Shi, S.P., ... & Yang, Y.P. (2015). Effect of a multi-level education intervention model on knowledge and attitudes of accidental injuries in rural children in Zunyi, Southwest China. International Journal of Environmental Research and Public Health, 12(4), 3903-3914.

**Notes**

a. High risk of bias in the randomization process, high risk of bias due to missing outcome data, high risk of bias in the measurement of the outcome, and some concerns on the selection of the reported results (Cochrane Risk of Bias-2).

# Part 4 – Combined basic swimming skills training (in water) and water safety training (out-of-water)

## Comparison 4.1. An educational programme combining basic swimming skills training and water safety skills with out-of-water training vs no educational programme for water safety skills

| N° of studies | Study design | Risk of bias | Inconsistency | Indirectness | Imprecision | Other considerations | Number of participants | | Effect | | Certainty |
| --- | --- | --- | --- | --- | --- | --- | --- | --- | --- | --- | --- |
|  |  |  |  |  |  |  | Intervention | Control | Relative (95%CI) | Absolute (95%CI) |  |
| Risk of death from drowning | | | | | | | | | | | |
| 1^1^ | Observational: retrospective cohort study | serious^a^ | not serious | not serious | not serious^b^ | none | 1/57834 (0.0%) | 77/102636 (0.1%) | aRR: 0.07 (0.02 to 0.31) | 0 fewer per 1000 (from 0 fewer to 0 fewer) | Moderate |
| Drowning mortality rate (per 100.000 person-years) at 4 years old | | | | | | | | | | | |
| 1^1^ | Observational: retrospective cohort study | very serious^c^ | not serious | not serious | very serious^d^ | none | --/--^e^ | --/--^e^ | Rate ratio: 0.55 (0.03 to 9.17) | - | Very low |
| Drowning mortality rate (per 100.000 person-years) at 5 years old | | | | | | | | | | | |
| 1^1^ | Observational: retrospective cohort study | very serious^c^ | not serious | not serious | very serious^d^ | none | --/--^e^ | --/--^e^ | Rate ratio: 0.24 (0.01 to 3.99) | - | Very low |
| Drowning mortality rate (per 100.000 person-years) at 6 years old | | | | | | | | | | | |
| 1^1^ | Observational: retrospective cohort study | very serious^c^ | not serious | not serious | very serious^d^ | none | --/--^e^ | --/--^e^ | Rate ratio: 0.17 (0.01 to 2.83) | - | Very low |
| Drowning mortality rate (per 100.000 person-years) at 7 years old | | | | | | | | | | | |
| 1^1^ | Observational: retrospective cohort study | very serious^c^ | not serious | not serious | very serious^d^ | none | --/--^e^ | --/--^e^ | Rate ratio: 0.17 (0.02 to 2.10) | - | Very low |
| Drowning mortality rate (per 100.000 person-years) at 8 years old | | | | | | | | | | | |
| 1^1^ | Observational: retrospective cohort study | very serious^c^ | not serious | not serious | very serious^d^ | none | --/--^e^ | --/--^e^ | Rate ratio: 0.12 (0.01 to 2.10) | - | Very low |
| Drowning mortality rate (per 100.000 person-years) at 9 years old | | | | | | | | | | | |
| 1^1^ | Observational: retrospective cohort study | very serious^c^ | not serious | not serious | very serious^d^ | none | --/--^e^ | --/--^e^ | Rate ratio: 0.20 (0.01 to 3.57) | - | Very low |
| Drowning mortality rate (per 100.000 person-years) at 10 years old | | | | | | | | | | | |
| 1^1^ | Observational: retrospective cohort study | very serious^c^ | not serious | not serious | very serious^d^ | none | --/--^e^ | --/--^e^ | Rate ratio: 0.72 (0.03 to 16.02) | - | Very low |
| Drowning mortality rate (per 100.000 person-years) at 11 years old | | | | | | | | | | | |
| 1^1^ | Observational: retrospective cohort study | very serious^c^ | not serious | not serious | very serious^d^ | none | --/--^e^ | --/--^e^ | Rate ratio: 3.79 (0.08 to 190.76) | - | Very low |
| Drowning mortality rate (per 100.000 person-years) at 12 years old | | | | | | | | | | | |
| 1^1^ | Observational: retrospective cohort study | very serious^c^ | not serious | not serious | very serious^d^ | none | --/--^e^ | --/--^e^ | Rate ratio: 6.00 (0.12 to 302.41) | - | Very low |
| Water safety skills: swimming ability (Scale from 0 to 15), after 8 weeks of training | | | | | | | | | | | |
| 1^2^ | Experimental: RCT | very serious^f^ | not serious | not serious | serious^g^ | none | 61 | 48 | - | MD: 2.80 higher (-- to --)^h^ (p < 0.05) | Very low |
| Water safety skills: swimming ability (Scale from 0 to 15), after 12 weeks of training | | | | | | | | | | | |
| 1^2^ | Experimental: RCT | very serious^f^ | not serious | not serious | serious^g^ | none | 61 | 48 | - | MD: 4.97 higher (-- to --)^h^ (p < 0.05) | Very low |
| Water safety skills: water recovery (Scale from 1 to 12), after 8 weeks of training | | | | | | | | | | | |
| 1^2^ | Experimental: RCT | very serious^f^ | not serious | not serious | serious^g^ | none | 61 | 48 | - | MD: 1.45 higher (-- to --)^h^ (p < 0.05) | Very low |
| Water safety skills: water recovery (Scale from 1 to 12), after 12 weeks of training | | | | | | | | | | | |
| 1^2^ | Experimental: RCT | very serious^f^ | not serious | not serious | serious^g^ | none | 61 | 48 | - | MD: 3.00 higher (-- to --)^h^ (p < 0.05) | Very low |
| Water safety skills: jump and swim (Scale from 1 to 12), after 8 weeks of training | | | | | | | | | | | |
| 1^2^ | Experimental: RCT | very serious^f^ | not serious | not serious | serious^g^ | none | 61 | 48 | - | MD: 0.31 higher (-- to --)^h^ (p > 0.05) | Very low |
| Water safety skills: jump and swim (Scale from 1 to 12), after 12 weeks of training | | | | | | | | | | | |
| 1^2^ | Experimental: RCT | very serious^f^ | not serious | not serious | serious^g^ | none | 61 | 48 | - | MD: 1.33 higher (-- to --)^h^ (p < 0.05) | Very low |
| Water safety behaviour: deck behaviour (Scale from 1 to 12), after 8 weeks of training | | | | | | | | | | | |
| 1^2^ | Experimental: RCT | serious^i^ | not serious | not serious | serious^g^ | none | 61 | 48 | - | MD: 0.33 lower (-- to --)^h^ (p > 0.05) | Low |
| Water safety behaviour: deck behaviour (Scale from 1 to 12), after 12 weeks of training | | | | | | | | | | | |
| 1^2^ | Experimental: RCT | serious^i^ | not serious | not serious | serious^g^ | none | 61 | 48 | - | MD: 0.2 higher (-- to --)^h^ (p > 0.05) | Low |
| Water safety behaviour: drowning rescues ever performed (SwimSafe vs non-swimmers) | | | | | | | | | | | |
| 1^3^ | Observational: retrospective cohort study | very serious^j^ | not serious | not serious | serious^k^ | none | 95/3890 (2.4%) | 2/3943 (0.1%) | RR: 48.15 (11.88 to 195.19) | - | Very low |
| Water safety behaviour: drowning rescues performed (SwimSafe vs natural swimmers) | | | | | | | | | | | |
| 1^3^ | Observational: retrospective cohort study | very serious^j^ | not serious | not serious | very serious^d^ | none | 95/3890 (2.4%) | 91/3924 (0.1%) | RR: 1.05  (0.79 to 1.40) | - | Very low |
| Water safety behaviour: rescues performed in previous month (SwimSafe vs natural swimmers) | | | | | | | | | | | |
| 1^3^ | Observational: retrospective cohort study | very serious^j^ | not serious | not serious | very serious^d^ | none | 29/3890 (0.7%) | 19/3924 (0.5%) | RR: 1.54 (0.86 to 2.74) | - | Very low |
| Water safety behaviour: rescues performed in previous year (SwimSafe vs natural swimmers) | | | | | | | | | | | |
| 1^3^ | Observational: retrospective cohort study | very serious^j^ | not serious | not serious | very serious^d^ | none | 90/3890 (3.1%) | 79/3924 (2.0%) | RR: 1.15 (0.85 to 1.55) | - | Very low |
| Water safety behaviour: drowning rescue rate (/1000) in previous month (SwimSafe vs natural swimmers), children aged 6-8 years | | | | | | | | | | | |
| 1^3^ | Observational: retrospective cohort study | very serious^j^ | not serious | not serious | very serious^l^ | none | 6/1322 (0.0%) | --/--^e^ | Rate ratio: 1.07 (-- to --)  (p > 0.05)^m^ | - | Very low |
| Water safety behaviour: drowning rescue rate (/1000) in previous month (SwimSafe vs natural swimmers), children aged 9-11 years | | | | | | | | | | | |
| 1^3^ | Observational: retrospective cohort study | very serious^j^ | not serious | not serious | very serious^l^ | none | 18/2096 (0.0%) | --/--^e^ | Rate ratio: 1.98 (-- to --)  (p > 0.05)^m^ | - | Very low |
| Water safety behaviour: drowning rescue rate (/1000) in previous month (SwimSafe vs natural swimmers), children aged 12-14 years | | | | | | | | | | | |
| 1^3^ | Observational: retrospective cohort study | very serious^j^ | not serious | not serious | very serious^l^ | none | 5/472 (0.0%) | --/--^e^ | Rate ratio: 1.24 (-- to --)  (p > 0.05)^m^ | - | Very low |
| Water safety behaviour: drowning rescue rate (/1000) in previous year (SwimSafe vs natural swimmers), children aged 6-8 years | | | | | | | | | | | |
| 1^3^ | Observational: retrospective cohort study | very serious^j^ | not serious | not serious | very serious^l^ | none | 17/1322 (0.0%) | --/--^e^ | Rate ratio: 1.20 (-- to --)  (p > 0.05)^m^ | - | Very low |
| Water safety behaviour: drowning rescue rate (/1000) in previous year (SwimSafe vs natural swimmers), children aged 9-11 years | | | | | | | | | | | |
| 1^3^ | Observational: retrospective cohort study | very serious^j^ | not serious | not serious | very serious^l^ | none | 61/2096 (0.0%) | --/--^e^ | Rate ratio: 1.27 (-- to --)  (p > 0.05)^m^ | - | Very low |
| Water safety behaviour: drowning rescue rate (/1000) in previous year (SwimSafe vs natural swimmers), children aged 12-14 years | | | | | | | | | | | |
| 1^3^ | Observational: retrospective cohort study | very serious^j^ | not serious | not serious | very serious^l^ | none | 12/472 (0.0%) | --/--^e^ | Rate ratio: 0.72 (-- to --)  (p > 0.05)^m^ | - | Very low |
| Water safety behaviour: 3 or more entries into a body of water, over 48 hours (SwimSafe vs natural swimmers) | | | | | | | | | | | |
| 1^4^ | Observational: retrospective cohort study | very serious^n^ | not serious | not serious | serious^o^ | none | 346/3523 (9.8%) | 300/3523 (8.5%) | RR: 1.15 (1.00 to 1.34) | - | Very low |
| Water safety behaviour: 2 entries into a body of water, over 48 hours (SwimSafe vs natural swimmers) | | | | | | | | | | | |
| 1^4^ | Observational: retrospective cohort study | very serious^n^ | not serious | not serious | not serious | none | 1692/3523 (48.0%) | 1773/3523 (50.3%) | RR: 0.95 (0.91 to 1.00) | - | Low |
| Water safety behaviour: 1 entry into a body of water, over 48 hours (SwimSafe vs natural swimmers) | | | | | | | | | | | |
| 1^4^ | Observational: retrospective cohort study | very serious^n^ | not serious | not serious | not serious | none | 356/3523 (10.1%) | 384/3523 (10.9%) | RR: 0.93 (0.81 to 1.06) | - | Low |
| Water safety behaviour: no entries into a body of water, over 48 hours (SwimSafe vs natural swimmers) | | | | | | | | | | | |
| 1^4^ | Observational: retrospective cohort study | very serious^n^ | not serious | not serious | not serious | none | 1129/3523 (32.0%) | 1066/3523 (30.3%) | RR: 1.06 (0.99 to 1.14) | - | Low |
| Water safety behaviour: water entries for playing or swimming, over 48 hours (SwimSafe vs natural swimmers) | | | | | | | | | | | |
| 1^4^ | Observational: retrospective cohort study | very serious^n^ | not serious | not serious | serious^d^ | none | 19/3523 (0.5%) | 11/3523 (0.3%) | RR: 1.73 (0.82 to 3.62) | - | Very low |
| Water safety behaviour: recreational water entries without adult supervision, over 48 hours (SwimSafe vs natural swimmers) | | | | | | | | | | | |
| 1^4^ | Observational: retrospective cohort study | very serious^n^ | not serious | not serious | serious^b^ | none | 30/31 (96.8%) | 11/15 (73.3%) | RR: 1.32 (0.97 to 1.80) | - | Very low |
| Cost-effectiveness (cost($Int) per death averted) | | | | | | | | | | | |
| 1^1^ | Observational: retrospective cohort study | serious^a^ | not serious | not serious | not serious | none | 57834 | 102636 | - | 3009 higher  (1813 to 19796 higher) | Moderate |
| Cost-effectiveness (cost($Int) per DALY averted) | | | | | | | | | | | |
| 1^1^ | Observational: retrospective cohort study | serious^a^ | not serious | not serious | not serious | none | 57834 | 102636 | - | 85 higher (51 to 561 higher) | Moderate |

**References:**

1. Rahman, F., Bose, S., Linnan, M., Rahman, A., Mashreky, S., & Haaland, B. (2012). Cost-effectiveness of an injury and drowning prevention program in Bangladesh. Pediatrics, 130(6), e1621-e1628.
2. Asher, K.N., Rivara, F.P., Felix, D., Vance, L., & Dunne, R. (1995). Water safety training as a potential means of reducing risk of young children’s drowning. Injury Prevention, 1(4), 228–233.
3. Mecrow, T.S., Rahman, A., Linnan, M., Scarr, J., Mashreky, S.R., Talab, A. & Rahman, A.K.M.F. (2015a). Children reporting rescuing other children drowning in rural Bangladesh: a descriptive study. Injury Prevention, 21(e1), e51–55.
4. Mecrow, T.S., Linnan, M., Rahman, A., Scarr, J., Mashreky, S.R., Talab, A. & Rahman, A.K.M.F. (2015b). Does teaching children to swim increase exposure to water or risk-taking when in the water? Emerging evidence from Bangladesh. Injury Prevention, 21(3), 185–188.

**Notes**

a. Moderate risk of bias due to confounding (ROBINS-I).
b. While the number of events is low, we decided not to rate down for imprecision due to the very large sample size.
c. Serious risk of bias due to confounding (ROBINS-I).
d. Downgraded by 2 levels due to a low number of events, and a large variability of results.
e. Number of participants not available per age group, so unable to calculate mortality rate.
f. High risk of bias in the randomization process, high risk of bias due to missing outcome data, high risk of bias in the measurement of the outcome, and some concerns in the selection of the reported results (Cochrane Risk of Bias-2).
g. Limited sample size.
h. 95% confidence interval was not reported in the study and could not be calculated due to multiple comparisons.
i. Some concerns on the randomization process, and some concerns on the selection of the reported results (Cochrane Risk of Bias-2).
j. Serious risk of bias in the measurement of the outcomes, moderate risk of bias due to confounding, and moderate risk of bias in the selection of the reported results (ROBINS-I).

k. Low number of events.
l. Downgraded by 2 levels for imprecision due to a low number of events and a lack of data, prohibiting us to assess whether the 95%CI contained both appreciable harm and benefit.
m. 95% confidence interval was not reported in the study and could not be calculated due to missing data. P value as reported by the study authors.
n. Serious risk of bias in the measurement of the outcomes, and moderate risk of bias due to confounding (ROBINS-I).
o. Large variability of the results

## Comparison 4.2. A 12-week educational programme combining basic swimming skills training and water safety skills with out-of-water training vs an 8-week educational programme combining basic swimming skills training and water safety skills with out-of-water training

| N° of studies | Study design | Risk of bias | Inconsistency | Indirectness | Imprecision | | Other considerations | | Number of participants | | | Effect | | | Certainty |
| --- | --- | --- | --- | --- | --- | --- | --- | --- | --- | --- | --- | --- | --- | --- | --- |
|  |  |  |  |  |  |  |  |  | Intervention | | Control | Relative (95%CI) | Absolute (95%CI) | |  |
| Water safety skills: Swimming ability (Scale from 0 to 15), at the end of the intervention | | | | | | | | | | | | | | | |
| 1^1^ | Experimental: RCT | very serious^a^ | not serious | not serious | serious^b^ | none | | 61 | | 48 | | - | MD: 0.60 (-- to --)^c^ (p > 0.05) | Very low | |
| Water safety skills: Swimming performance in domain "water recovery" (Scale from 1 to 12), at the end of the intervention | | | | | | | | | | | | | | | |
| 1^1^ | Experimental: RCT | very serious^a^ | not serious | not serious | serious^b^ | none | | 61 | | 48 | | - | MD: 0.01 (-- to --)^c^ (p > 0.05) | Very low | |
| Water safety skills: Swimming performance in domain " water recovery" (Scale from 1 to 12), at 12 weeks follow-up | | | | | | | | | | | | | | | |
| 1^1^ | Experimental: RCT | very serious^a^ | not serious | not serious | serious^b^ | none | | 61 | | 48 | | - | MD: 1.18 (-- to --)^c^ (p > 0.05) | Very low | |
| Water safety skills: Swimming performance in domain "jump and swim" (Scale from 1 to 12), at the end of the intervention | | | | | | | | | | | | | | | |
| 1^1^ | Experimental: RCT | very serious^a^ | not serious | not serious | serious^b^ | none | | 61 | | 48 | | - | MD: 0.77 (-- to --)^c^ (p > 0.05) | Very low | |
| Water safety skills: Swimming performance in domain "jump and swim" (Scale from 1 to 12), at 12 weeks follow-up | | | | | | | | | | | | | | | |
| 1^1^ | Experimental: RCT | very serious^a^ | not serious | not serious | serious^b^ | none | | 61 | | 48 | | - | MD: 0.88 (-- to --)^c^ (p > 0.05) | Very low | |
| Water safety behaviour: Deck behaviour (Scale from 1 to 12), at the end of the intervention | | | | | | | | | | | | | | | |
| 1^1^ | Experimental: RCT | serious^d^ | not serious | not serious | serious^b^ | none | | 61 | | 48 | | - | MD: 0.26 (-- to --)^c^ (p > 0.05) | Low | |
| Water safety behaviour: Deck behaviour (Scale from 1 to 12), at 12 weeks follow-up | | | | | | | | | | | | | | | |
| 1^1^ | Experimental: RCT | serious^d^ | not serious | not serious | serious^b^ | none | | 61 | | 48 | | - | MD: 0.13 (-- to --)^c^ (p > 0.05) | Low | |

**References**

1. Asher, K.N., Rivara, F.P., Felix, D., Vance, L., & Dunne, R. (1995). Water safety training as a potential means of reducing risk of young children’s drowning. Injury Prevention, 1(4), 228–233.

**Notes**

a. High risk of bias in the randomization process, high risk of bias due to missing outcome data, high risk of bias in the measurement of the outcome, and some concerns in the selection of the reported results (Cochrane Risk of Bias-2).
b. Limited sample size.
c. 95% confidence interval was not reported in the study and could not be calculated due to multiple comparisons.
d. Some concerns on the randomization process, and some concerns on the selection of the reported results (Cochrane Risk of Bias-2).
